# Granulocyte-colony stimulating factor gene therapy as a novel therapeutics for stroke in a mouse model

**DOI:** 10.1186/s12929-020-00692-5

**Published:** 2020-10-30

**Authors:** Janet M. Menzie-Suderam, Jigar Modi, Hongyaun Xu, Andrew Bent, Paula Trujillo, Kristen Medley, Eugenia Jimenez, Jessica Shen, Michael Marshall, Rui Tao, Howard Prentice, Jang-Yen Wu

**Affiliations:** 1 Department of Biomedical Sciences, Charles E. Schmidt College of Medicine, Florida Atlantic University, Boca Raton, FL 33431 USA; 2 Program in Integrative Biology, Florida Atlantic University, Boca Raton, FL 33431 USA; 3 Complex Systems and Brain Sciences, Florida Atlantic University, Boca Raton, FL USA; 4 College of Medicine, New York University, New York, NY 10003 USA; 5 AEURA Trust, 2525 Arapahoe Ave E4-138, Boulder, CO 80302 USA

**Keywords:** Granulocyte-colony stimulating factor (g-CSF), Gene therapy, Endoplasmic reticulum stress apoptosis, Mitochondrial dynamics, Autophagy, Transient global ischemia

## Abstract

**Background:**

Global ischemia is the resulting effect of a cardiopulmonary arrest (CPA). Presently there is no effective treatment to address neurological deficits in patients who survived a CPA. Granulocyte-colony stimulating factor is a growth factor (G-CSF) with a plethora of beneficial effects, including neuroprotection. Clinical application of human G-CSF (hG-CSF) is limited due to its plasma half-life of 4 h. Therefore, novel approaches need to be investigated that would (1) enable prolonged manifestation of hG-CSF and (2) demonstrate G-CSF efficacy from studying the underlying protective mechanisms of hG-CSF. In our previous work, we used the self-complementary adeno-associated virus (stereotype2: scAAV2) as a vector to transfect the hG-CSF gene into the global ischemic brain of a mouse. As an extension of that work, we now seek to elucidate the protective mechanisms of hG-CSF gene therapy against endoplasmic reticulum induced stress, mitochondrial dynamics and autophagy in global ischemia.

**Method:**

A single drop of either AAV-CMV-hG-CSF or AAV-CMV-GFP was dropped into the conjunctival sac of the Swiss Webster mouse’s left eye, 30–60 min after bilateral common artery occlusion (BCAO). The efficacy of the expressed hG-CSF gene product was analyzed by monitoring the expression levels of endoplasmic reticulum stress (ER), mitochondrial dynamics and autophagic proteins over 4- and 7-days post-BCAO in vulnerable brain regions including the striatum, overlying cortex (frontal brain regions) and the hippocampus (middle brain regions). Statistical analysis was performed using mostly One-Way Analysis of variance (ANOVA), except for behavioral analysis, which used Repeated Measures Two-Way ANOVA, post hoc analysis was performed using the Tukey test.

**Results:**

Several biomarkers that facilitated cellular death, including CHOP and GRP78 (ER stress) DRP1 (mitochondrial dynamics) and Beclin 1, p62 and LC3-ll (autophagy) were significantly downregulated by hG-CSF gene transfer. hG-CSF gene therapy also significantly upregulated antiapoptotic Bcl2 while downregulating pro-apoptotic Bax. The beneficial effects of hG-CSF gene therapy resulted in an overall improvement in functional behavior.

**Conclusion:**

Taken together, this study has substantiated the approach of sustaining the protein expression of hG-CSF by eye drop administration of the hG-CSF gene. In addition, the study has validated the efficacy of using hG-CSF gene therapy against endoplasmic reticulum induced stress, mitochondrial dynamics and autophagy in global ischemia.

## Introduction

Cardiopulmonary arrest is one of the major causes of death and disability in the world. One significant consequence of cardiopulmonary arrest is global ischemia. Although there have been improved emergency treatments (such as therapeutic hypothermia), the survival rate for out-of-hospital cardiac arrest patients remains approximately 8% for the past three decades [1]. Reduced blood flow to the brain, as observed in ischemia, results in dysfunction of several critical organelles, such as the endoplasmic reticulum (ER) and mitochondria. The defective function of the ER and or the mitochondria may lead to autophagy and imminently, neuronal death. The ER synthesizes and folds proteins for physiological purposes. Any condition, such as ischemia that interferes with the standard folding of proteins in the ER results in ER stress [2]. In order to overcome the initial ER stress signal, the ER elicits a response called the Unfolded Protein Response (UPR) which is executed by three ER transmembrane stress sensors: inositol-requiring protein-1(IRE1), protein kinase RNA (PKR)-like ER kinase (PERK), and activating transcription factor 6 (ATF6). In non-stressed cells, the three ER sensors are kept inactive through binding to the ER chaperone glucose-regulated protein 78 (GRP78)/immunoglobulin-binding protein (Bip), but in stressed cells, the ER sensors activate respective intracellular pathways to alleviate ER stress [3, 4]. However, if the cell is subjected to prolonged ER stress that it cannot alleviate, transcriptional factor C/EBP homologous protein (CHOP), a pro- apoptotic protein, is induced by one or more of the three ER stress sensors [5, 6]. CHOP is the most important mediator of ER stress-induced apoptosis [7]. It modulates Bcl-2 family members’ transcription by inhibiting transcription of the anti-apoptotic protein, Bcl-2 [8] while enhancing the transcription of the several pro-apoptotic proteins, including Bim and Bax [9–12].

The balance between mitochondrial fission and fusion plays an integral role in mitochondrial physiology. Several essential proteins are involved in maintaining mitochondrial dynamics, including dynamin-related protein 1 (DRP1; fission protein) and optic atrophy 1 (OPA1; fusion protein). Ischemia disrupts mitochondrial dynamics by downregulating fusion proteins, such as OPA1; which promotes the mitochondrial fission phenotype [13–15]. Disturbances in ER and mitochondrial function during an ischemic injury elicit an autophagic condition and ultimately cell death. Autophagy is a well- established contributor to cellular homeostasis, which involves the fusion of injured organelles with a lysosome (forming an autophagosome) for enzymatic degradation [16]. While autophagy is an important phenomenon for cell homeostasis, excessive autophagy leads to autophagic cell death, and the activation of apoptotic signaling pathways [17]. Acute and severe ischemia causes excessive autophagy demonstrated by an increase in Beclin 1 (autophagy initiator) [18]. To address the numerous complex pathways of cerebral ischemia, therapeutic agents should be multipotent.

G-CSF is a hematopoietic growth factor that is FDA approved for the treatment of neutropenia. G-CSF stimulates growth and differentiation of hematopoietic stem cells [19], and both G-CSF and its receptor are located in the CNS [20]. G-CSF has been shown to pass through the blood–brain barrier by receptor-mediated endocytosis, leading to a reduction in infarct volume in rat models of acute stroke [21]. Several studies have demonstrated G-CSF to be neuroprotective, angiogenic, neurogenic and improve motor function [22, 23]. A phase IIb trial has also tested the safety of G-CSF [24]. Despite G-CSF's vast therapeutic potential, its half-life is only 4 h, limiting the translational use of G-CSF in the clinic. To overcome this limitation, we need to re-evaluate G-CSF’s efficacy, using an approach that could produce a lasting effect of exogenous G-CSF protein. In our previous work we demonstrated that an eye drop administration of human G-CSF (hG-CSF) in a replication-deficient, self-complementary adeno-associated virus (AAV) vector (scAAV2-CMV-hG-CSF) would enable a sustained expression of the exogenous hG-CSF protein.[25]. Therefore, in our present study, we seek to elucidate the protective mechanisms of hG-CSF gene therapy; pertaining to ischemic pathology; including mitochondrial dysfunction, ER stress and autophagy.

## Materials and methods

### Animals

Adult male Swiss Webster mice (weighing 40–50 g, Taconic Farm, USA) were kept cages in a room with controlled light cycles (12 h/12 h light/dark) at a constant temperature (21 ± 1 °C). Food and water were provided ad libitum except during the behavioral assessment. All animal procedures were carried out following the guidelines for care and animal’s use and were approved by the institutional animal care and use committee (IACUC) of Florida Atlantic University, Boca Raton.

### Transient bilateral carotid artery occlusion (BCAO) to Induce global cerebral ischemia

The BCAO method used to induced global ischemia was the same as previously published [26]. Briefly, mice were weighed and initially anesthetized with 2.5% isoflurane in O_2_/air mixture using a vaporizer. During surgery, in maintaining anesthesia, isoflurane concentration was reduced to 0.5% and an intra-peritoneal (IP) injection of ketamine hydrochloride (40 mg/kg body weight; Putney) and xylazine hydrochloride (2 mg/kg body weight; Vedco) cocktail was administered. Ventrally, a sagittal incision was made in the mouse neck (~ 1 cm length). Both salivary glands were separated to visualize the underlying common carotid arteries (CCAs). Dissections were made between the sternocleidomastoid and the sternohyoid muscles parallel to the trachea, to expose the CCAs. The CCAs were carefully separated from the respective vagal nerves and accompanying veins. A lose 5-0 silk suture loop was made around each CCA, which was used to gently lift the CCAs while the CCAs were clamped with microaneurysm clips. The CCAs remained occluded for 30 min after which micro clips were removed with subsequent reperfusion. During the surgery, core body temperature was maintained at 37.0 ± 0.5 °C by controlled water circulating heating pad. Local cerebral blood flow (LCBF) was monitored in the cerebral cortex of either the left or right hemisphere by positioning a Doppler laser probe (Laser Doppler flowmeter LDF; Perimed Inc., Cleveland, OH, USA) over the area of the middle cerebral artery (MCA) on the left or right of the skull. Only mice with a LDF signal reduced to a minimum of 50% of the baseline signal were included in the study. Mice with incomplete reperfusion (i.e., < 90% recovery of CBF compared to baseline CBF, n = 4) were also excluded from the study.

### Design and construct of expression vectors

**Fig. 1:**
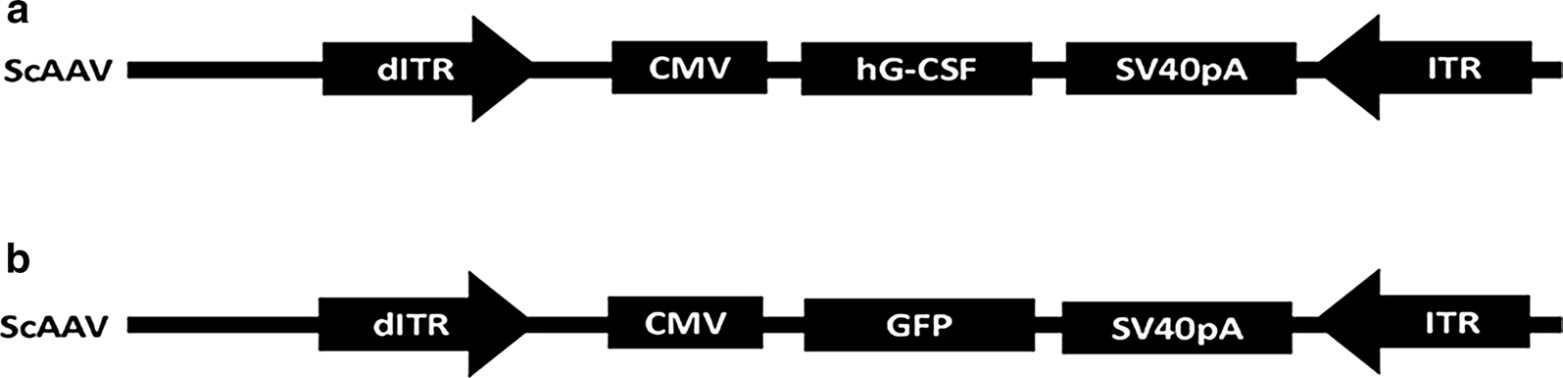
Schematic diagram of the vectors used in this investigation. The CMV promoter is incorporated into scAAV2 either **a** in conjunction with the hG-CSF cDNA open reading frame or **b** in conjunction with the GFP cDNA open reading frame. The SV40 polyA (SV40pA) sequence and the inverted terminal repeat sequence (ITR) are also incorporated into the scAAV vectors (dITR denotes the mutated ITR in the 5 prime end which enables generation of self-complementary AAV). Construction of the vectors is described in the methods section

The green fluorescent protein (GFP) -encoding plasmid used in this investigation (scAAV2-CMV-GFP; Fig. 1) was a gift from Dr. D. McCarty (Ohio State University, Columbus, OH, USA). The cDNA sequence for hG-CSF isoform B (accession number NM_172219.2) was amplified by PCR and sub-cloned into scAAV2-CMV-GFP by substituting the hG-CSF cDNA sequence for the GFP cDNA sequence. The resulting hG- CSF encoding plasmid containing the CMV promoter, the hG-CSF cDNA and the SV40 poly-adenylation sequence was thus termed scAAV2-CMV-hG-CSF (Fig. 1). Viral vectors were produced using scAAV2-CMV-hGCSF and scAAV-CMV-GFP at the Gene Therapy Vector Core, University of North Carolina (Chapel Hill, NC) and titers were determined by standard dot blot analysis.

### Mice groupings and scAAV treatment

Animals were randomly assigned to one of three group (sham operation control n = 16, BCAO n = 27or BCAO plus treatment, n = 21). The control animals underwent surgical procedures identical to BCAO, except that the CCAs were not occluded. The BCAO animals were given a single administration of either scAAV2-CMV-hG-CSF or scAAV2-CMV-GFP (3 × 109 pfu; 1.5 µl), to the eye sac of the left eye 30–60 min post BCAO/reperfusion, while sham-operated mice were given 1.5 µl saline to the left eye sac. There was a significant difference in LCBF between sham and the BCAO group (*p* < 0.001), but no significant difference in LCBF between the BCAO + AAV-CMV-GFP group and the BCAO + AAV-CMV-hG-CSF group (Additioanl file 1: Fig. S1). Animals were then euthanized at two time points of either 4 days (sham n = 10, BCAO + AAV-CMV-GFP n = 18, BCAO + AAV-CMV-hG-CSF n = 13) or 7 days (sham n = 6, BCAO + AAV-CMV-GFP n = 9, BCAO + AAV-CMV-hG-CSF n = 8).

### Quantitative reverse transcription polymerase chain reaction (qRT-PCR)

Total RNA was extracted from the brains of mice (Sham n = , AAV-CMV-GFP n = , AAV-CMV-hG-CSF n =) on day 4 with the RNeasy Mini Kit (Qiagen, CA, USA), according to the manufacturer’s directions. Samples were treated to remove DNA by using the Turbo DNase (Thermo Fisher Scientific). RNA was reverse transcribed utilizing a random pd(N)6 primer and Thermoscript RT-PCR system (Life Technologies). Quantitative reverse transcription real-time PCR analysis (qRT-PCR) of the viral transcripts was obtained as previously described [26]. qRT-PCR analysis for the mRNA of Bcl2, Bax, and GAD65 was performed. All respective primers are identified from the PubMed primer-Blast tool (https://blast.ncbi.nlm.nih.gov/) (Table 1). We generated a primer with antisense (AS) sequence (5′-AACTCGGGGGAGATCCCTTCCA, homemade) to hG-CSF mRNA (AS-hG-CSF) and sense (S) sequence (5″-ACTCTCTGGGCATCCCCT, S-hG-CSF). An hG-CSF—specific reverse transcription product using primer AS-hG-CSF was generated using reverse transcriptase. The primers used for G-CSF were specific to the human G-CSF RNA produced by the hG-CSF gene and not specific for mouse G-CSF RNA. Each sample was normalized for the relevant content with the housekeeping gene GAPDH. qRT-PCR was performed utilizing an Agilent Aria Mx real-time PCR system, SYBR green dye and analyzed with Aria Mx 1.5 software. Each assay was carried out with a minimum of three independent transfections while qRT-PCR assays were carried out in duplicates.

**Table 1:** PCR primer information

Genes	Primer-forward (5′–3′)	Primer-reverse (5′—3′)	PCR product length (bp)
hG-CSF	AACTCGGGGGAGATCCCTTCCA	ACTCTCTGGGCATCCCCT	142
GAD65	GCTCATCGCGTTCACATCAG	AGTAACCCTCCACCCCAAGC	300
BCL2	TGGGATGCCTTTGTGGAACT	GAGACAGCCAGGAGAAATCA	67
BAX	CCA CCAGCTCTGAACAGATC	CAGCTTCTTGGTGGAGGCAT	140
GAPDH	TCCATGACAACTTTGGCATCGTGG	GTTGCTGTTGAAGTCACAGGAGAC	366

A description of the forward and reverse primers used in the qRT-PCR analysis for the mRNAs, of hG-CSF, GAD65, BCL2, BAX and GAPDH (control)

### Infarct staining

Animals were deeply anesthetized by isoflurane (Phoenix) and decapitated, and then brains were rapidly removed. Using an adult mouse brain matrix (Matrix, Zivic Instruments), brains were sectioned coronally into six 2-mm coronal slices (2, 4, 6, 8, 10, and 12 mm from the frontal pole) and incubated for 5 min in a 2% (w/v) solution of 2,3,5-triphenyltetrazolium chloride (TTC; JT Baker, India) at 37 °C for staining followed by collecting samples for western blot [27].

### Western blot analysis

Mice brains were removed and quickly dissected on ice using a brain matrix. Sections of 2 mm coronal brain slices were divided into frontal (0–4 mm sections, which included the striatum and overlying cortex), middle (6–8 mm sections, which included the hippocampus and surrounding brain structures) and hind (10–12 mm sections, which included the cerebellum). The frontal and middle brain sections were used since the common carotid arteries supplied these areas. Western blot was done as described previously [28]. Briefly, brain sections were homogenized in RIPA buffer (25 mM Tris–HCl pH 7.6, 150 mM NaCl, 1% NP-40, 1% sodium deoxycholate, 0.1% SDS) containing 1% (*v*/*v*) mammalian protease inhibitor cocktail and 2% (*v*/*v*) phosphatase inhibitor cocktail (Sigma and Thermo Scientific, respectively). Homogenate was centrifuged at 12,000 rpm for 15 min at 4 °C. The supernatant was collected while the pellet discarded. Bradford protein assay determined-protein concentrations were then resolved by 12% sodium dodecyl sulfate-polyacrylamide gel electrophoresis and blotted to nitrocellulose membrane. Nitrocellulose membrane was then incubated overnight (4 °C) with the following primary antibodies: Abcam: GRP78, ATF4, p-IRE1, OPA1 (all at 1:1000), GAD65 (1:500) and XBP-1 (1:500); Cell Signaling: GAPDH (1:3000), Bcl2 (1:1000), AKT, p-AKT and Beclin 1 (all at 1:1000); Santa Cruz: CHOP/GADD153 (1:100), Bax (1:500) and hG-CSF (1;500); Millipore: DRP1 (1:1000) and Imgenex: ATF6 (1:500); followed by three washes with Tris-buffered saline containing 0.1% Tween-20 (TBS-T) and then incubated with fluorescence conjugated secondary antibodies, goat IRDye 800-conjugated anti-rabbit (1:15,000) or anti-mouse (1;15,000) (LI-COR Biosciences, Lincoln, NE, USA) for 2 h at RT. GAPDH was used as internal loading controls for whole cell lysate samples. The hG-CSF antibody (Santa Cruz, cat. # sc53292) was specific to human G-CSF protein produced by the human G-CSF gene. All antibodies with the respective company and catalog number are listed Additioanl file 3: Table S1) and a representative example of western whole blot result is shown (Additioanl file 2: Fig. S2). Fluorescent signals were detected with an LI-COR Odyssey Fc system. Densitometric analysis was done by Image Studio Lite version 5.1. Independent experiments were carried out in duplicates or triplicates.

### Histology

The histological analysis involved nissl staining and immunofluorescence and was carried out by a blinded observer to the study. Mice were perfused transcardially with phosphate-buffered saline (PBS: 50 mM NaCl, 10 mM Na-phosphate buffer, pH 7.4) followed by 4% paraformaldehyde. Brains were removed, post-fixed in 4% paraformaldehyde overnight at 4 °C, and cryopreserved with 30% sucrose, then freeze at − 80 °C in an anti-freeze solution (50% PBS, 20% ethylene glycol and 30% glycerol). Sections were further process using nissl staining or immunofluorescence.

### Nissl staining

For each animal, twelve serial sets of coronal brain sections (40 µm) were cut on Leica cryostat between 1.94 and -2.46 posterior to bregma. Mice brain sections were washed twice for 15 min in 0.1 M PBS and incubated in 0.5% cresyl violet staining solution for 10–15 min at room temperature. Following incubation, the sections were washed with distilled water and dehydrated gradually in ethanol (70%, 95% and 100%), then placed in xylene as a final processing step. Sections were cover slipped with mounting media. Ten consecutive sections (four sections for striatum and cortex and six sections for hippocampus) per brain were analyzed (Sham n = 4, AAV-CMV-GFP n = 6 and AAV-CMV-hG-CSF n = 5). Five non-overlapping visual fields (0.25 mm^2^) of the striatum and three non-overlapping visual fields (0.25 mm^2^) of the cortex were randomly selected, while two non-overlapping visual fields (0.25 mm^2^) of CA3, CA2 and CA1 were randomly selected for statistical analysis. Images were captured with an epifluorescence microscope, converted to 8-bit grayscale, threshold, size excluded by setting a minimum pixel area size was set, watershed to separate close cells and automated counted using NIH Image J. Neurons with round and palely stained nuclei were considered surviving cells, whereas shrunken cells with pyknotic nuclei were considered dying cells.

### Immunofluorescence

Free-floating sections were blocked in blocking buffer (10% normal goat serum, 0.3% TritonX-100, 0.3 M glycine, 1:40 goat anti-mouse Fab fragment in TBS) for 1 h. at room temperature. Sections were incubated with rabbit anti-GAD, 1:200 (laboratory of Dr. Jang Yen Wu), mouse anti-Chat, 1:500; (Santa Cruz: sc-55557) or rabbit anti-NeuN, 1:500 (Millipore Sigma: MAB377) overnight at 4 °C followed by incubation with anti-rabbit Alexa Fluor 488-conjugated, anti-mouse Alexa Fluor 488-conjugated (1:2000; Invitrogen), or anti-rabbit Rhodamine Red-conjugated, 1:500 (Jackson ImmunoReach AB2338022) for 2 h at RT. After washing with TBS-T (3 times, 5 mins each), the sections were mounted on saline-coated microscope slides with Prolong Gold antifade reagent (Invitrogen). Ten consecutive sections per brain were analyzed for GAD, with four sections for cortex and six sections for the CA3 and CA1 (n = 4 per group). Four non-overlapping visual fields (0.32 mm^2^) of the cortex, two non-overlapping visual fields (0.32 mm^2^) of the hippocampus’s CA3 and CA1 were selected at random for statistical analysis. Another set of three consecutive sections per brain were analyzed for ChAT and NeuN colocalization in the forebrain (two 0.32 mm^2^ nonoverlapping visual fields). Fluorescence signals were detected by a Zeiss LSM700 confocal laser scanning microscope. Collected images were quantified for GAD and CHAT immuno-positive (GAD^**+**^ and CHAT^**+**^ respectively) neurons by converting images into 8-bit grayscale, threshold, size excluded by setting a minimum pixel area size and automated counted using NIH Image J software. Negative controls were performed by omitting the primary antibody (data not shown).

### Corner test

All behavioral paradigms were performed by an observer blinded to the study. The corner test, which determines an animal’s asymmetric direction of turning when encountering a corner, is used to indicate brain injury. We used an experimental corner setup composed of two boards (with dimensions of 30 × 20 × 1 cm^3^) arranged to form a 30° corner; a small opening was left along the joint between the two boards. The mouse was placed 12 cm from the corner and allowed to walk into the corner so that the vibrissae on both sides of the animal's face made contact with the two boards simultaneously. Before the BCAO procedure, we conducted behavior tests (stratification) on all mice to screen for mice with no turning asymmetry (n ≥ 16). Each mouse took part in ten trials, after which we calculated the percentage of turns to each side, recording only those turns involving full rearing along one of the boards. This stratification procedure excludes mice with a pre-test score of 80–100% asymmetric turns (n = 4).

### Locomotor activity

The locomotor activity test was determined by a force-plate actometer (Model FPA-I; West Lafayette, IN, USA) as described [29]. The actometer consists of a force-sensitive plate at a 200 Hz resolution, a sound-attenuation chamber, a computerized data-acquisition board (42 × 42 cm) and analysis system software (FPA 1.10.01). In the test, the animal was placed on the board plate for a 60-min period, and data of tracing the movements were automatically stored on the hard drive for offline analysis. Locomotor activity was performed on mice before BCAO and 4- and 7-days post-BCAO.

### Statistical tests

Data were analyzed using GraphPad Prism 7.0 software (GraphPad, San Diego, CA, USA). Behavioral measurements were analyzed by two-way ANOVA repeated measures, followed by Tukey’s post hoc test. All other analyses were performed with one-way ANOVA, followed by Tukey’s post hoc test. Differences in p-values were considered significant if *p* < 0.05. Data are represented as the mean ± SEM independent experiments were performed in duplicates or triplicates. In figures *p* < 0.05 is labeled as * or ^#^*p* < 0.01 is labeled as ** or ^##^ and *p* < 0.001 is labeled as *** or ^###^based on the respective comparison.

## Results

### Surgery

The mortality rate (4%) was low in our 30 min BCAO model of global ischemia. Mice were randomly assigned to receive either AAV-CMV-GFP or AAV-CMV-hG-CSF pre-BCAO. All animals subjected to BCAO showed a significant reduction in LCBF. Mice designated to receive AAV-CMV-GFP showed a 62% ± 5% drop in baseline LCBF while AAV-CMV- hG-CSF designated mice showed a 69% ± 8% drop in baseline LCBF (FAdditioanl file 1: Fig. S1 During reperfusion, mice (1%) with < 90% LCBF were excluded from the study. Sham operated mice showed no significant fall in LCBF from baseline (Additioanl file 1: Fig. S1). In our previous study [23] we demonstrated that a 50–55% fall in LCBF of 90 min-MCAO (middle cerebral artery occlusion) was sufficient to elicit cerebral ischemia. Interestingly, our current study with 30 min-BCAO showed a greater drop in LCBF (62–69%) than our previous work [23] with 90 min-MCAO. The greater fall in LCBF of BCAO is indicative of occluding bilaterally arteries contrasted to the unilateral occluded artery in the MCAO and supports evidence of the effectiveness of the BCAO model to induce global cerebral ischemia [30].

### Expression of the human G-CSF (hG-CSF) gene via administration of adeno- associated viral vector to the BCAO mouse eye sac

**Fig. 2:**
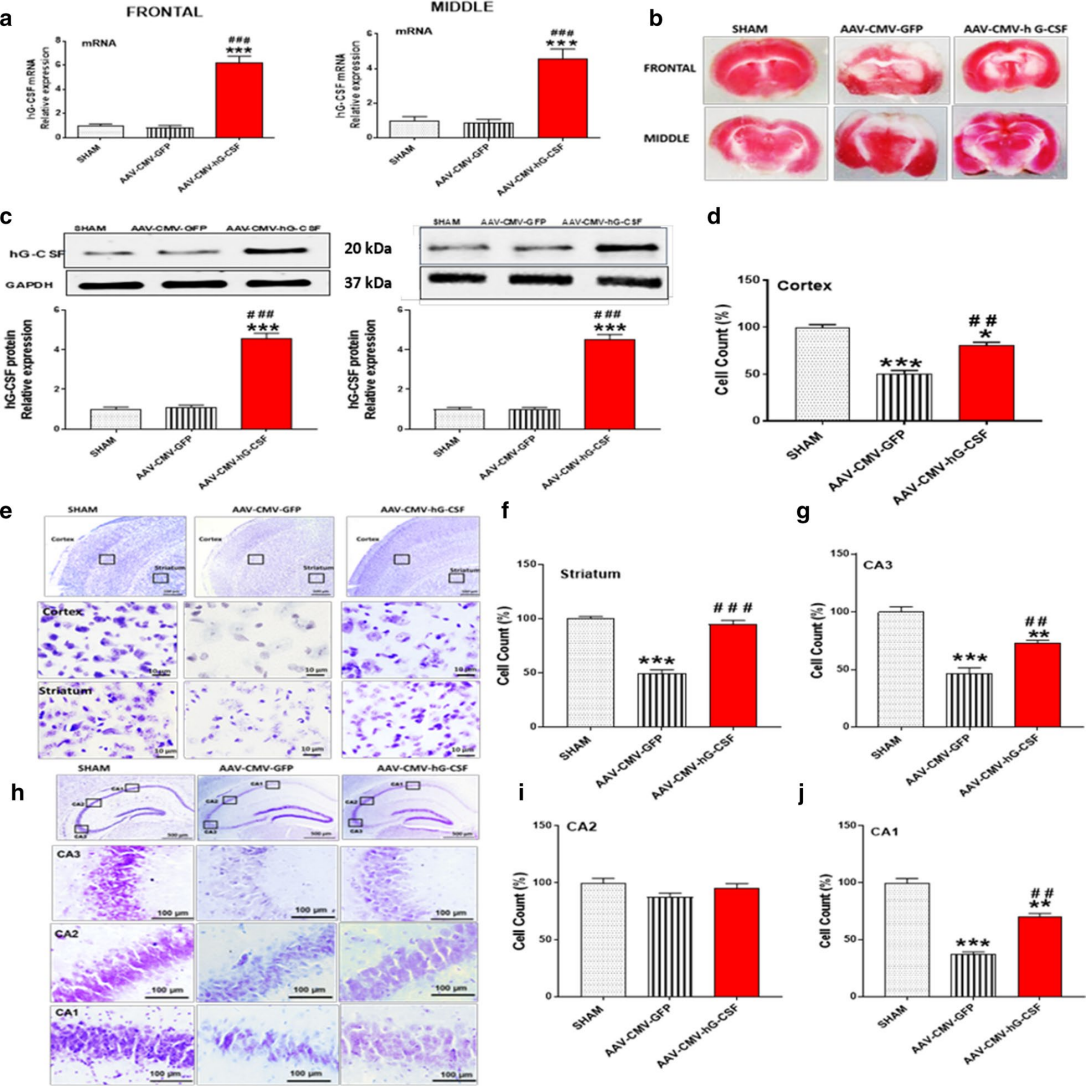
Demonstration of hG-CSF gene expression via adeno-associated viral administration, four days post BCAO. Mice were given AAV-CMV-hG-CSF or AAV-CMV-GFP (1.5ul) by eye-drop in the left eye following 30 min of BCAO. Sham non-ischemic mice received no virus. All analysis was carried out on the frontal and middle brain sections. **a** qRT-PCR confirms the expression of mRNA hG-CSF levels in the frontal and middle brain sections **c**. Western blot validated the significant translation of mRNA hG-CSF to the hG-CSF protein. **b** TTC representation of infarcted regions. White areas indicative of infarction **d–j** Nissl staining of the frontal brain sections (cortex and striatum) and middle brain sections (hippocampus). Representative photomicrograph of **e** cortex and striatum and of the **h** hippocampal CA3, CA2 and CA1. Quantitative nissl stain analysis of the **d** cortex, **f** striatum, **g** CA3, **i** CA2 and **(j)** CA1 is shown (sham n = 4, AAV-CMV-GFP n = 6 and AAV-CMV-hG-CSF n = 5). Statistical analysis was performed by One Way ANOVA, with Tukey as post hoc test and expressed as means ± S.E.M; *p < 0.05 /**p < 0.01/***p < 0.001 versus Sham (n = 5) group while ^##^*p* < 0.01/^###^*p* < 0.001 versus AAV-CMV-GFP- treated group. (n = 7 animals for AAV-CMV-hG-CSF and AAV-CMV-GFP- treated groups

Genes carried by an AAV vector are delivered as one single-bolus dose in most gene therapies. To evaluate gene therapy using eye drop delivery, we administered a single dose of scAAV2-CMV-hG-CSF (3 × 109 pfu; 1.5 µl) into the left eye 30–60 min post-reperfusion. Then performed qRT-PCR (left hemisphere) for hG-CSF mRNA expression and western blot (right hemisphere) for hG-CSF protein expression in sham-operated, AAV-CMV-GFP group and the AAV-CMV-hG-CSF group. After administering the viral vectors, we observed a dramatic increase, (≥ 5-fold) 4 days post-viral transfection of both hG-CSF mRNA and protein expression in the AAV-CMV-hG-CSF treated group compared to both sham mice and the AAV-CMV-GFP treated mice (n = 8/group; ***p < 0.001 and ^###^*p* < 0.001; Fig. 2a, c). Therefore, our data demonstrated that we successfully expressed exogenous hG-CSF via the hG- CSF gene’s viral administration through the BCAO mice eye sac. Moreover, these data support a stable scAAV2 vector and its cDNA after gene therapy. Next, we investigated any effect the expressed hG-CSF protein may have on gross brain morphology by performing TTC on the frontal and middle brain sections. We detected a clear representation of less infarction in the brains of AAV-CMV-hG-CSF mice over the AAV-CMV-GFP mice (Fig. 2b). Although we did not quantify infarct volume in this study, the infarct representation and qualitative observation is supported by several evidence that G-CSF significantly reduces infarct volume in cerebral ischemia [22, 23, 31]

### hG-CSF gene therapy attenuates cellular death

Using cresyl violet (staining DNA and RNA nucleotides) to stain nissl bodies, we observed that hG-CSF gene expression reduced cellular loss, 4 days post-BCAO, by 50% in the striatum (^###^*p* < 0.001; Fig. 2e, f) and by 30% in overlaying cortex (^##^*p* < 0.01; Fig. 2e, d). In addition, the hG-CSF protection was also observed in the cornu ammonis 3 (CA3; 73%) and cornu ammonis 1 (CA1; 70%) regions of the hippocampus (^##^*p* < 0.01; Fig. 2h–J). There were no significant differences amongst the groups in the cornu ammonis 2 (CA2) region (*p* = 0.1320).

### hG-CSF gene therapy decreases apoptosis by upregulating the Bcl2 family members

**Fig. 3:**
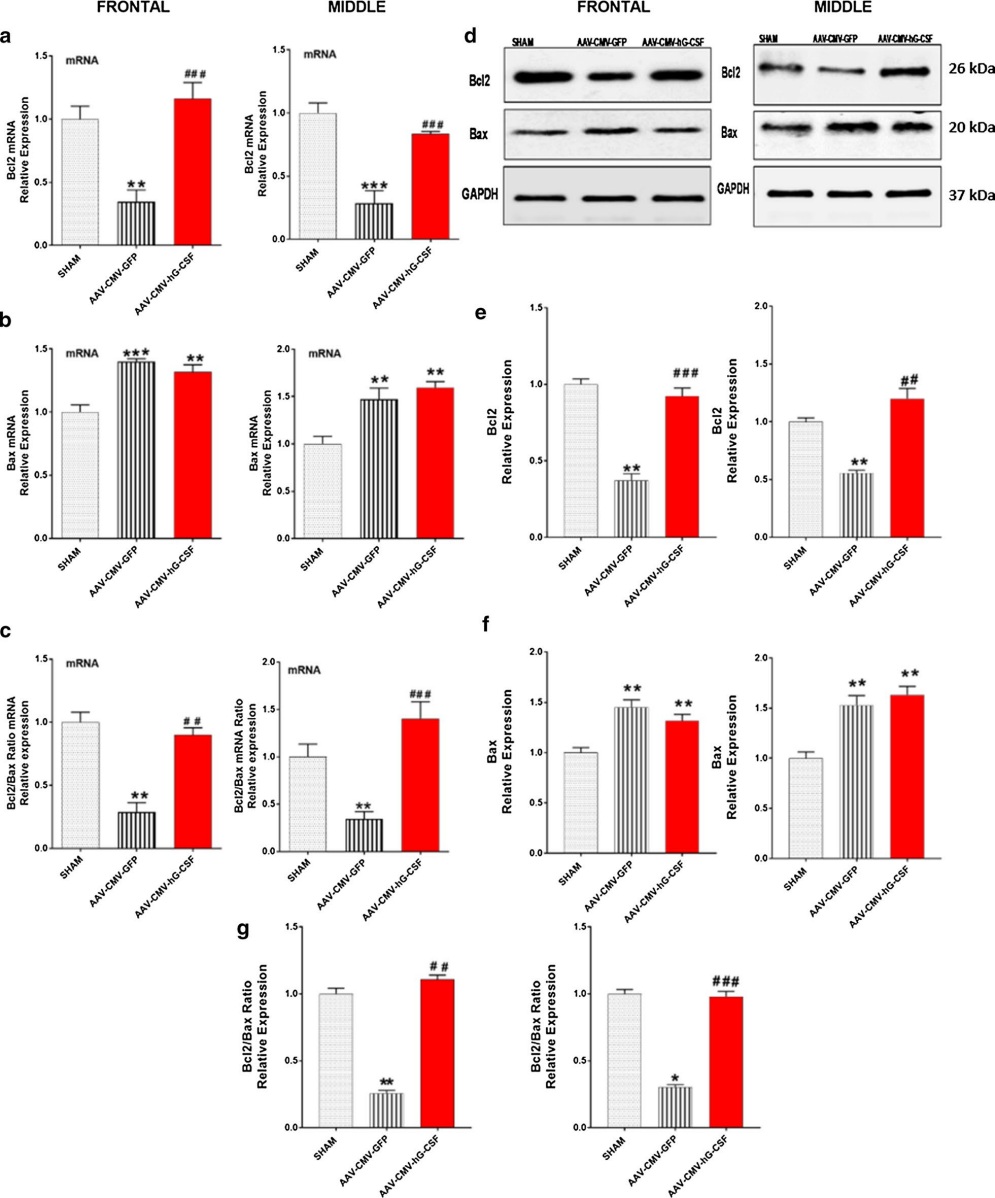
Effect of hG-CSF gene therapy on apoptotic modulators: Bcl2 and Bax. **a–c** qRT-PCR validating the expression levels of Bcl2, Bax and Bcl2:Bax mRNA ratio respectively, 4 days post BCAO. **d–f** Western blot analysis of the translated mRNA into protein expression of Bcl2, Bax. **g** Ratio of Bcl2:Bax protein expression. Representative densitometry for respective Western blots is also shown. Statistical analysis was performed by One Way ANOVA, with Tukey as post hoc test and expressed as means ± S.E.M; *p < 0.05/**p < 0.01/***p < 0.001 versus Sham group while ^#^*p* < 0.05 /^##^*p* < 0.01/^###^*p* < 0.001 versus AAV-CMV-GFP- treated group. (n = 6 animals/group)

Bcl2 family members such as Bax and Bak are major pro-apoptotic proteins while on the other hand, Bcl2 and Bcl-xL are major anti-apoptotic proteins [32]. We investigated the effect of hG-CSF gene therapy on the expression of Bcl2 and Bax. Brain samples of sham (n = 4), AAV-CMV-GFP (n = 5) and AAV-CMV-hG-CSF treated (n = 6) mice, 4 days after BCAO were harvested. qRT-PCR and western blotting were performed on the frontal and middle brain regions. The level of Bax mRNA and protein expression in both brain sections of AAV-CMV-GFP treated mice were significantly increased compared to sham mice (**p < 0.01/***p < 0.001; Fig. 3b, f). In contrast, Bcl2 mRNA and protein expression were markedly reduced in the AAV-CMV-GFP mice versus sham-operated mice (**p < 0.01/***p < 0.001; Fig. 3a, e). Ischemia induced changes of Bcl2 and Bax levels resulted in a significant reduction in the Bcl2:Bax ratio of both brain sections in AAV-CMV- GFP treated mice compared to the sham group (Fig. 3c, g). The reduction of Bcl2:Bax ratio was effectively increased by hG-CSF treatment in both brain sections (Fig. 3c, g), evident by the increased Bcl2 and reduced Bax mRNA (Fig. 3a, b) and protein expressions (Fig. 3d–f) in the AAV-CMV-hG-CSF mice. This demonstrated that the expressed exogenous hG-CSF gene reverses the levels of pro-apoptotic proteins and anti-apoptotic proteins, in favor of cell survival.

### The effect of hG-CSF gene therapy on apoptosis induced by endoplasmic reticulum stress

Based on the importance of GRP78 and CHOP in ER stress-induced apoptosis [5, 6], we decided to investigate the effect of hG-CSF gene therapy on GRP78 and CHOP expression as well as examine which one of the three ER stress pathways is modulated by hG-CSF gene treatment.

### CHOP and GRP78

**Fig. 4:**
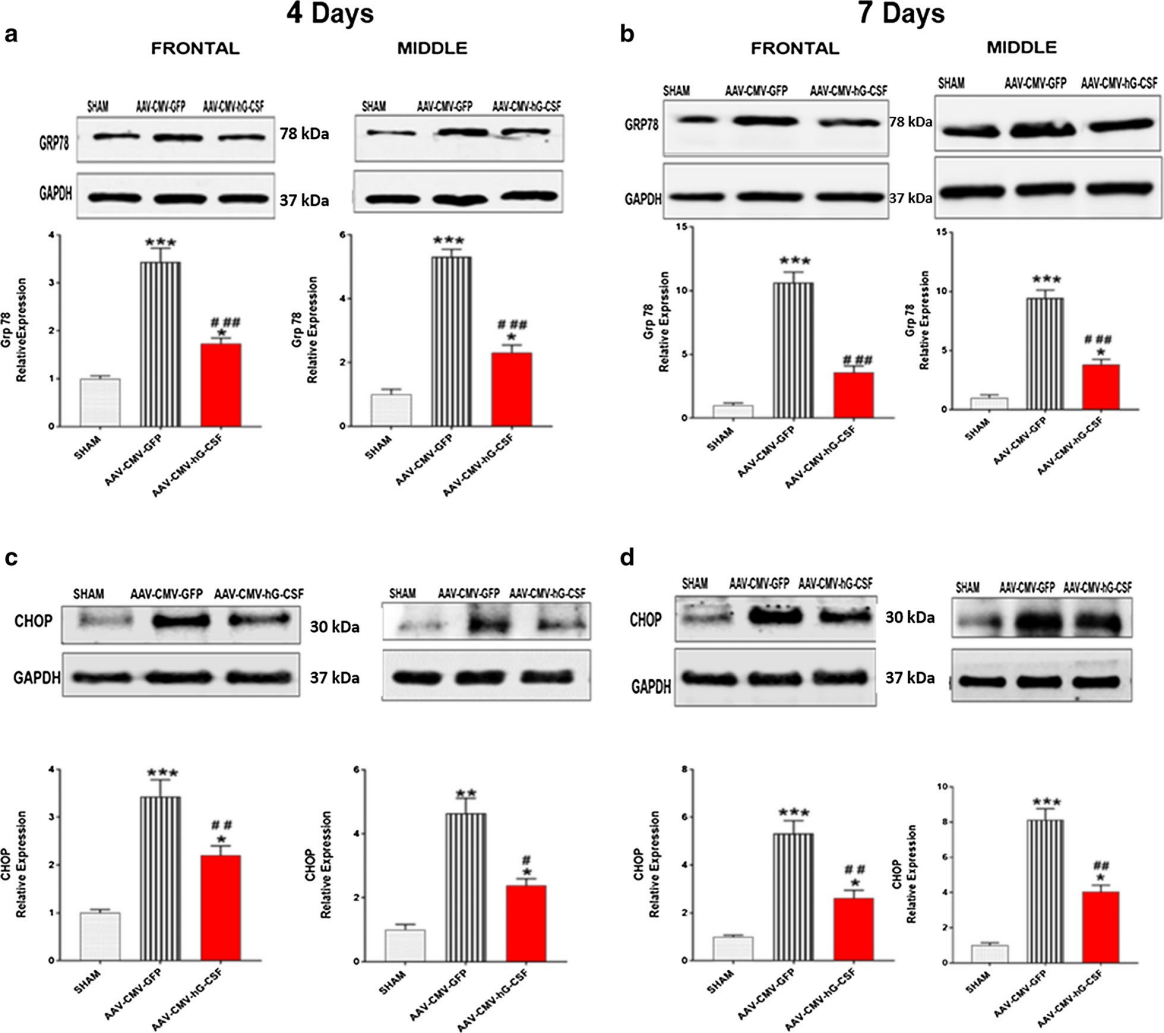
Effect of hG-CSF gene therapy on endoplasmic reticulum stress. GRP78/BiP is an ER chaperone molecule as well as an ER intraluminal stress sensor, while the CHOP is a molecular hallmark of ER stress. **a, b** Western blot analysis of GRP78 in the frontal and middle brain sections of sham, AAV-CMV-GFP and AAV-CMV-hG-CSF treated mice at **a** 4- and **b** 7-days post-BCAO. **c, d** Similarly, western blot analysis of CHOP at **c** 4- and **d** 7-days. Statistical analysis was performed by One Way ANOVA, with Tukey as post hoc test and expressed as means ± S.E.M; *p < 0.05/**p < 0.01/***p < 0.001 versus Sham group while ^#^*p* < 0.05/^##^*p* < 0.01/^###^*p* < 0.001 versus AAV-CMV-GFP- treated group (n = 6 animals/group)

The expression of GRP78 and CHOP was significantly upregulated in both brain sections of the AAV-CMV-GFP group, at 4- (n = 7 per group; Fig. 4a, c) and 7- (n = 5 per group **p < 0.01/***p < 0.001; Fig. 4b, d) days compared to sham mice. hG-CSF gene therapy significantly reduced the expression level of both GRP78 (^##^*p* < 0.01; Fig. 4a, b) and CHOP (^#^*p* < 0.05/^##^*p* < 0.01; Fig. 4c, d) to 50% compared to the AAV-CMV-GFP group. Since GRP78 is the ER luminal stress sensor, the reduction of GRP78 by hG-CSF gene treatment indicates that the constitutive expression of hG-CSF at 4- and 7-days post- BCAO reduces ER stress in the global ischemic brain. Also, the attenuation of CHOP (the downstream executive effector of ER stress apoptosis) in the AAV-CMV-hG-CSF treated mice at these two-time points definitively provided evidence that hG-CSF gene treatment ameliorates ER stress.

### Upstream ER regulatory pathways of CHOP

**Fig. 5:**
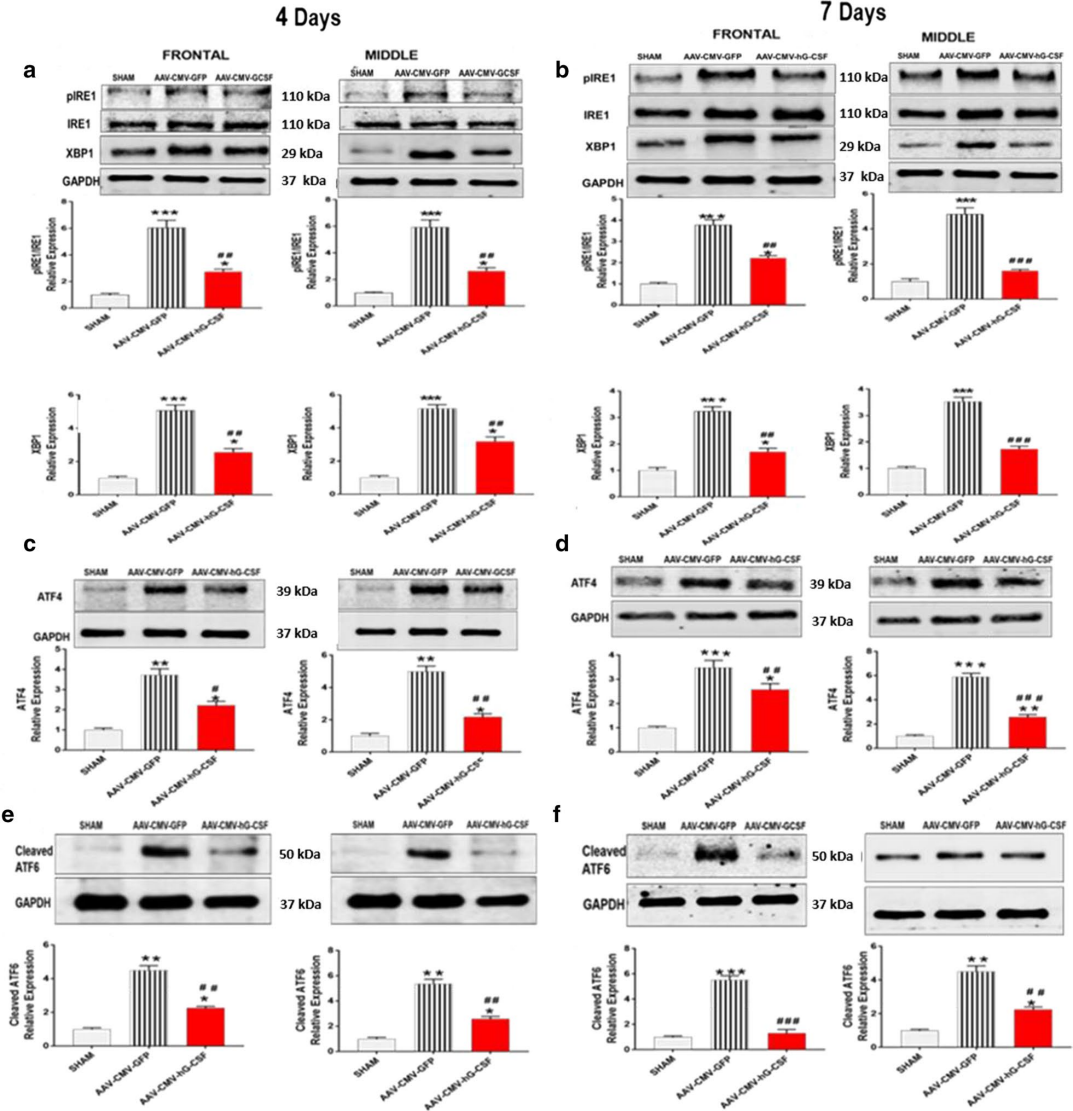
Effect of hG-CSF gene therapy on the IRE1 and PERK and ATF6 pathway. **a, b** Western blot analysis of IREI and its downstream player, in the frontal and middle brain sections of sham, AAV-CMV-GFP and AAV-CMV-hG-CSF treated mice at **a** 4- and **b** 7-days post-BCAO (n = 6 animals/group). **c, d** Western blot analysis of the downstream player in the PERK pathway, ATF4, in the frontal and middle brain sections of sham, AAV-CMV-GFP and AAV-CMV-hG-CSF treated mice at **c** 4- and **d** 7- days post-BCAO and **e, f** western blot analysis of cleaved ATF6 **e** 4- and **f** 7-days post-BCAO. Statistical analysis was performed by One Way ANOVA, with Tukey as post hoc test and expressed as means ± S.E.M; *p < 0.05/**p < 0.01/***p < 0.001 versus Sham group while ^#^*p* < 0.05/^##^*p* < 0.01/^###^*p* < 0.001 versus AAV-CMV-GFP- treated group. (n = 7 animals/group)

The ER stress pathways upstream of CHOP are the IRE1-PERK- and ATF6- pathways. Each pathway has a key factor; X-BP1, ATF4 and cleaved ATF6, respectively, that regulates the transcription of CHOP. We investigated the effect that hG-CSF gene treatment may have on X-BP1, ATF4 and cleaved ATF6 at 4- and 7-days post-BCAO in the frontal and middle brain regions. Our data indicated that the protein levels of, X-BP1, ATF4 and cleaved ATF6 were drastically increased in the AAV-CMV-GFP mice compared to sham mice at 4- and 7-days post-BCAO (**p < 0.01/***p < 0.001; Fig. 5a–f). Treatment with hG-CSF gene significantly decreased the expression level of X-BP1, ATF4 and cleaved ATF6, at 4- (^#^*p* < 0.01/^##^*p* < 0.001; Fig. 5a, c, e) and 7- (^##^*p* < 0.01/^###^*p* < 0.001; Fig. 5b, d, f) compared to GFP control. In addition, at 4- and 7-days, phosphorylated IRE1 (pIRE1: upstream to X-BP1) was downregulated in the AAV-CMV-hG-CSF group compared to the AAV-CMV-GFP group (^##^*p* < 0.01/^###^*p* < 0.001 Fig. 5a, b).

### Caspase 12 and phosphorylated protein kinase B (pAKT)

**Fig. 6:**
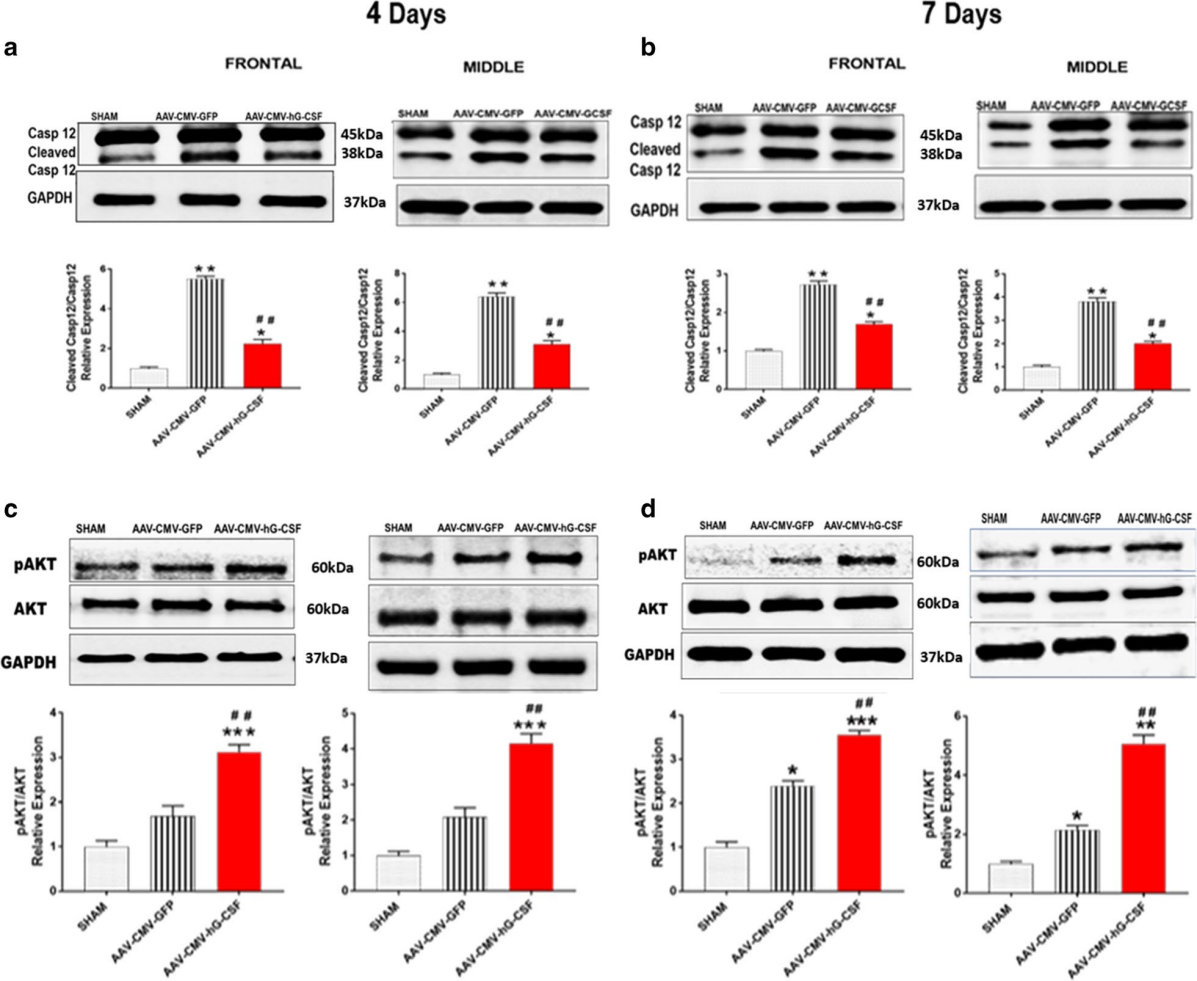
Effect of hG-CSF gene therapy on the Caspase 12 and pAKT. **a, b** Western blot analysis of cleaved caspase 12 (cleaved casp 12) **a** 4- and **b** 7-days post-BCAO. **c, d** similarly western blot analysis of pAKT in the frontal and middle brain sections of sham, AAV-CMV-GFP and AAV-CMV-hG-CSF treated mice at **c** 4- and **d** 7- days post-BCAO. Statistical analysis was performed by One Way ANOVA, with Tukey as post hoc test and expressed as means ± S.E.M; *p < 0.05/**p < 0.01/***p < 0.001 versus Sham group while ^#^*p* < 0.05/##*p* < 0.01/^###^*p* < 0.001 versus AAV-CMV-GFP- treated group. (n = 7 animals/group)

Caspase 12, a pro-death protein localized at the ER membrane, is activated via cleavage during ER stress [33]. Our data showed that hG-CSF gene therapy modulated the expression of cleaved caspase 12 by downregulating the level of cleaved caspase 12, 4- and 7-days post-BCAO (^##^*p* < 0.01; Fig. 6a, b). The major pathway that hG-CSF mediates its protective effect is via G-CSF receptor-activation of the phosphoinositide 3-kinase (PI3K)/AKT pathway [34]. We observed overexpression of pAKT (Ser-473), by hG- CSF gene compared to sham and GFP control group (***p < 0.001 and ^###^*p* < 0.001, respectively; Fig. 6c, d). This result suggests that hG-CSF gene expression works at least in part via the PI3K/AKT pathway. Our data is supported by Schneider and colleagues [34]. Using the AKT blocker (LY294002), Schneider and colleagues inhibit pAKT expression in the presence of G-CSF. They also showed that the inhibition by LY294002B reduced G-CSF protection, evident by the increase in caspase 3/7 activity [34]. Interestingly, pAKT was induced in the GFP control mice, which indicates that even in the absence of therapy, the injured brain attempts to protect itself.

### hG-CSF gene therapy modulates mitochondrial dynamics and autophagy

**Fig. 7:**
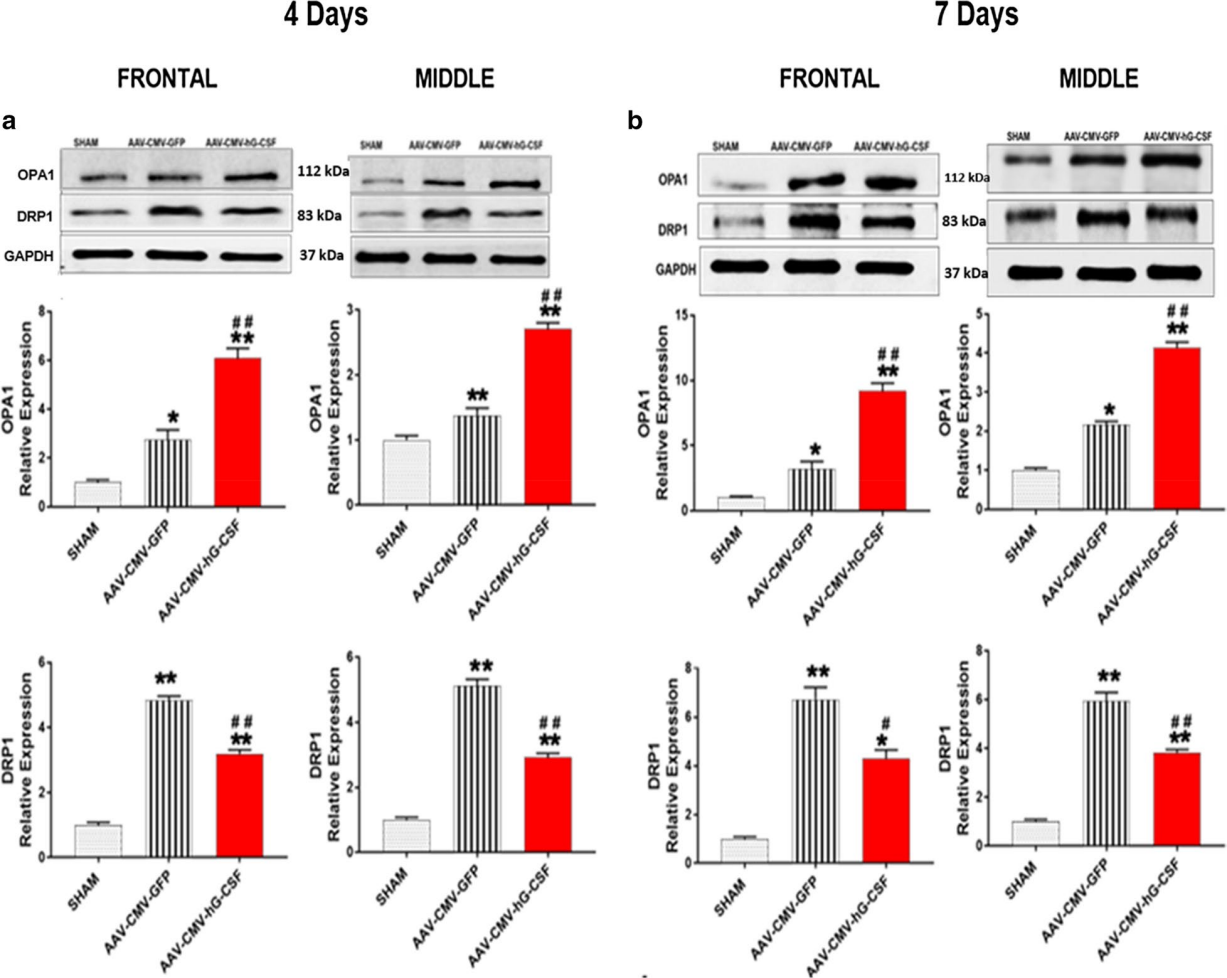
hG-CSF gene therapy effect on mitochondrial dynamics. Western blot of proteins that are involve in mitochondrial dynamics; OPA1 and DRP1 at **a** 4 days post- and **b** 7 days post- BCAO in the front and middle brain sections respectively. Respective densitometry indicates significance amongst the groups at 4 days and 7 days. Statistical analysis was performed by One Way ANOVA, with Tukey as post hoc test and expressed as means ± S.E.M; *p < 0.05 or **p < 0.001 versus Sham group while ^#^*p* < 0.05 or ^##^*p* < 0.001 versus AAV-CMV-GFP- treated group (Sham n = 5, AAV-CMV-GFP n = 6, AAV-CMV-GFP n = 6)

**Fig. 8:**
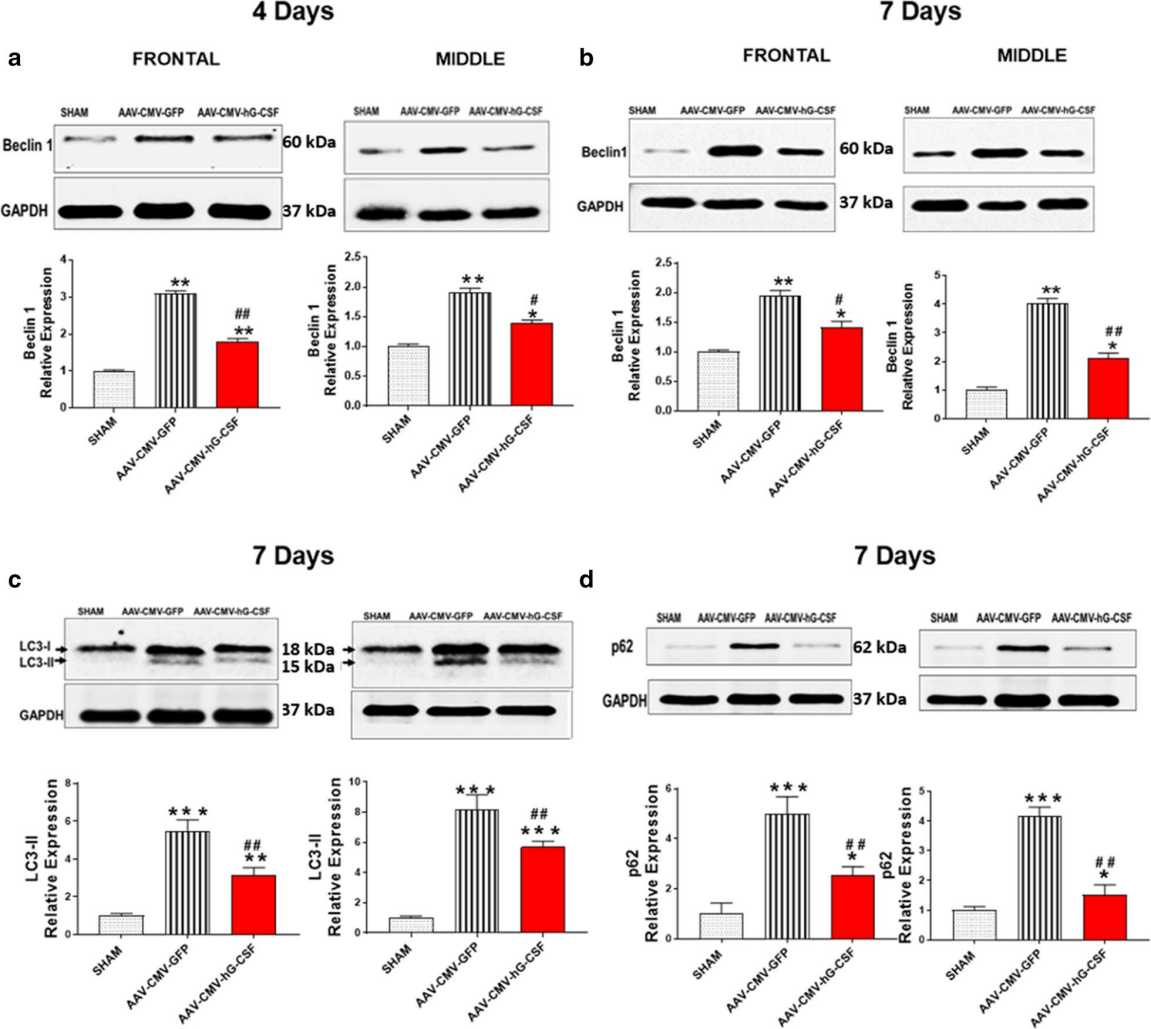
hG-CSF gene therapy effect on autophagy. **a, b** The expression level of the autophagy initiator protein; Beclin 1 is demonstrated **a** 4 and **b** 7-days post BCAO. **c, d** expression levels of the autophagosome marker, **c** LC3-ll and the autophagic substrate, **d** p62, 7 -days post BCAO. Respective densitometry of Beclin 1, LC3-ll and p62 are also shown. Statistical analysis was performed by One Way ANOVA, with Tukey as post hoc test and expressed as means ± S.E.M; *p < 0.05 or **p < 0.001 versus Sham group while ^#^*p* < 0.05 or ^##^*p* < 0.001 versus AAV-CMV-GFP- treated group (Sham n = 5, AAV-CMV-GFP n = 6, AAV-CMV-GFP n = 6)

To investigate the possible beneficial effect of hG-CSF gene therapy in transient global ischemia/reperfusion on mitochondrial dynamics, we observed the changes in mitochondrial fusion and fission proteins; OPA1 and DRP (respectively). During mitochondrial dysfunction, the mitochondria undergo fission (fragmentation), increasing DRP1. Our data from western blotting showed that global IR injury significantly increased the expression level of the mitochondrial fission protein, DRP1 (**p < 0.01 Fig. 7a, b), which is consistent with several reports [35, 36]. The upregulated DRP1 expression was attenuated by hG-CSF gene therapy at 4- (^##^*p* < 0.01; Fig. 7a) and 7- (^#^*p* < 0.05/^##^*p* < 0.01; Fig. 7b) days post BCAO. In conjunction with the reduced level of DRP1 by hG-CSF gene, hG-CSF gene therapy drastically potentiated the expression level of the fusion protein OPA1 compared to the control group treated with GFP (^##^*p* < 0.01; Fig. 7a, b). The marked increase of OPA1 and reduction of DRP1 by hG-CSF gene therapy counteract mitochondrial fission in transient global ischemic injury. Studies have reported the upregulation of Beclin 1, a cellular biomarker of autophagy, in ischemic brain injury [18]. Our data showed that hG-CSF gene transfer had a positive effect of reducing the level of Beclin 1 in the global ischemic brain (^#^*p* < 0.05/^##^*p* < 0.01; Fig. 8a, b). The cytosolic form of Microtubule-associated proteins (MAP)-light chain 3 (LC3-I) is converted to LC3-ll by phosphatidylethanolamine (PE) conjugate. LC3-ll is recruited to the autophagosome membrane and is therefore a key biomarker of autophagosome formation during autophagy [37, 38]. G-CSF gene therapy significantly decreased expression of LC3-Il, to 58% and 69% in frontal and middle brain regions respectively relative to GFP-treated BCAO, 7 days post BCAO (Fig. 8c). Additionally, the adaptor protein, p62 (sequestrome1: SQSTM1/p62), is an autophagic substrate and is degraded on autophagosome processing [39]. Therefore, the level of p62 is inversely related to autophagic activity [40, 41]. We observed a fourfold increase in the level of p62 in both the front and middle brain regions of the vehicle treated BCAO, 7 days post-BCAO, indicating reduced autophagic activity. However, G-CSF gene transfer significantly attenuated the p62 expression (Fig. 8d), indicating a return of autophagic activity.

### Effect of hG-CSF gene therapy on GABAergic neurons and cholinergic neurons

**Fig. 9:**
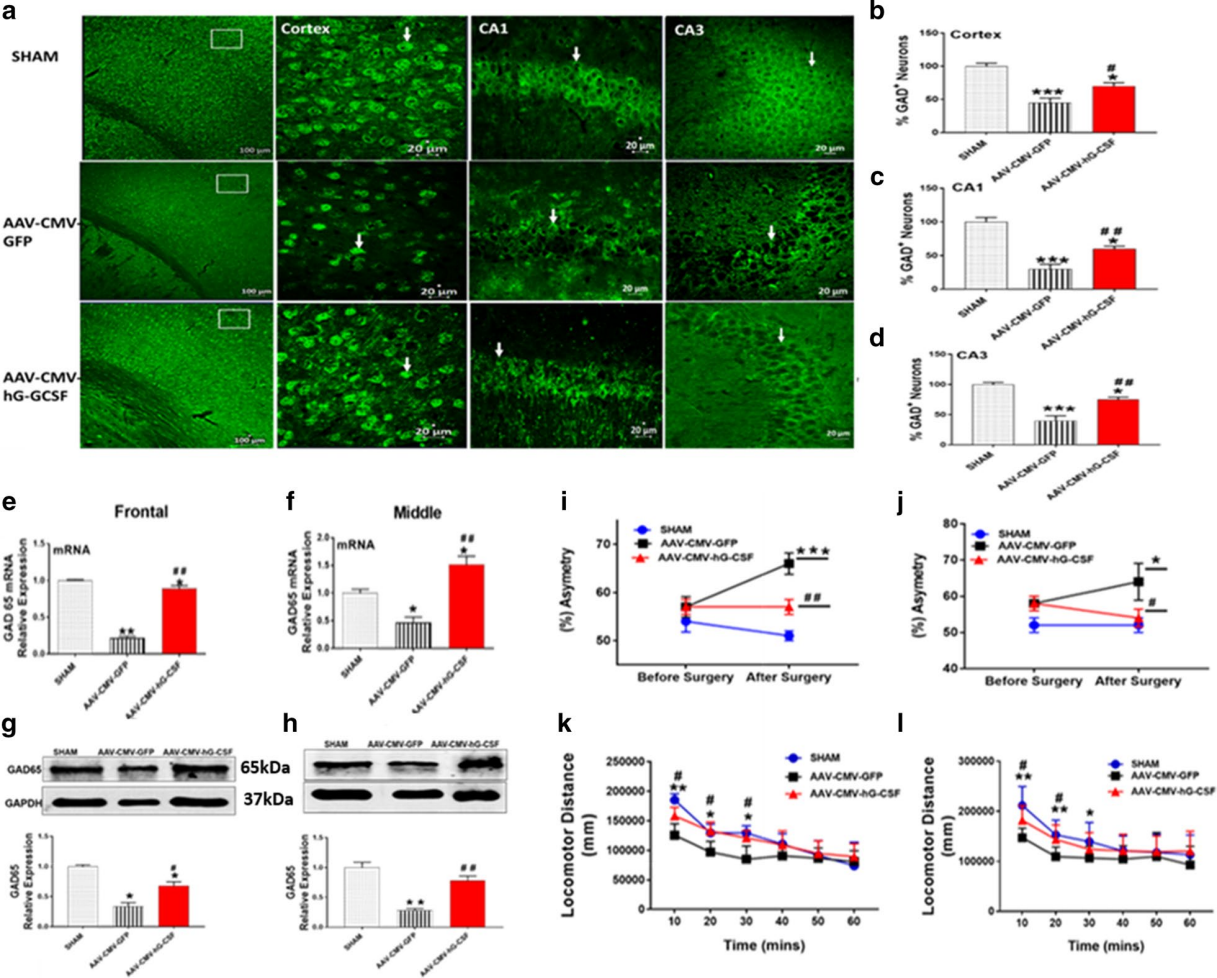
Effect of hG-CSF gene therapy on GABAergic neurons and functional behavior. **a** Photomicrograph of GAD expression in the cortex, CA1 and CA3 regions of the hippocampus at 4 days post BCAO. White arrow head indicates immunopositive expression of GAD. **b–d **Quantitative analysis of GAD expression in **b** cortex, and hippocampal **c** CA1 and **d** CA3. qRT-PCR and western analysis **e, f** mRNA and **g, h** protein of GAD65 expression level at 4 days post BCAO. **i–l** Behavioral assessment using **i, j** corner test and **k, l** locomotor activity **i, k** 4- and **j, l** 7-days respectively. Statistical analysis for behavioral analysis (Sham n = 16; AAV-CMV-GFP n = 27; AAV-CMV-hG-CSF n = 21) was performed by Two Way ANOVA repeated measures, with Tukey as post hoc test and expressed as means ± S.E.M; *p < 0.05/**p < 0.01/***p < 0.001 versus Sham group ^#^*p* < 0.05/^##^*p* < 0.01 versus AAV-CMV-GFP- treated group. All other analysis was performed with One Way ANOVA, with Tukey as post hoc test means ± S.E.M; *p < 0.05/**p < 0.01/***p < 0.001 versus Sham group while ^#^*p* < 0.05/ ^##^*p* < 0.01 versus AAV-CMV-GFP- treated group (n = 6 per group)

**Fig. 10:**
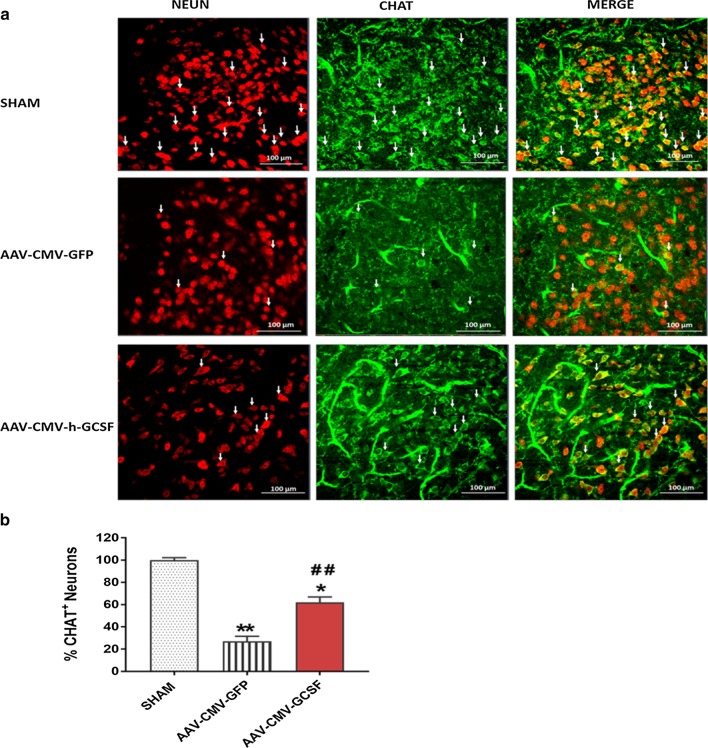
Effect of hG-CSF gene therapy on Cholinergic neurons. Expression of cholinergic neurons in the mouse basal forebrain 4 days post BCAO. **a** Photomicrograph of CHAT (Choline acetyltransferase: the enzyme that synthesize acetylcholine) expression in the basal forebrain at 4 days post BCAO. White arrow indicates immunopositive expression of CHAT neurons. **b** Quantitative analysis of CHAT expression in the basal forebrain. Statistical analysis was performed with One Way ANOVA, and Tukey as post hoc test. Means ± S.E.M; *p < 0.05/**p < 0.01 versus Sham group while ^##^*p* < 0.01 versus AAV-CMV-GFP- treated group (n = 3 per group)

Glutamic acid decarboxylase (GAD) exists as two isoforms, GAD65 and GAD67. GAD is enzyme in neurons that catalyzes the decarboxylation of glutamate to gamma-aminobutyric acid (GABA), the primary inhibitory neurotransmitter in the brain. Therefore, the cellular expression of GAD is indicative of GABAergic neurons. We observed that at 4 days post-BCAO, the levels of GAD immunopositive neurons were reduced in the cortex, and in the CA1 and CA3 hippocampal cornus of the AAV-CMV-GFP mice compared to sham (**p < 0.01/***p < 0.001; Fig. 9a–d). Treatment with hG-CSF gene successfully rescued GAD immunopositive neurons by 64% in the cortex (^#^*p* < 0.05; Fig. 9a, b), 50% in CA1 (^##^*p* < 0.01; Fig. 9g, c) and 53% in CA3 (^##^*p* < 0.01; Fig. 9g, d). Additionally, we observed a significant increase in GAD65 (the enzyme associated with GABA synaptic vesicles) mRNA and protein level in the AAV-CMV-hG-CSF treated mice compared to the AAV-CMV-GFP mice (^#^*p* < 0.05/^##^*p* < 0.01 Fig. 9e–h). This suggests that the beneficial effect that hG-CSF gene treatment provided to GABAergic neurons, could reduce the overall excitation by GABA-mediated inhibition and overall protection of the brain from ischemia. In addition to finding a beneficial effect of treatment with hG-CSF gene on GABAergic neurons in the cortex and hippocampus we observed a significant increase of cholinergic neurons as demonstrated by a higher number of ChAT positive neurons in the basal forebrain at 4 days post BCAO (Fig. 10).

### Effect of hG-CSF gene therapy determined by behavioral test

The corner test is used to assess neurological deficits in rodents and it is viewed as “more sensitive” amongst the five sensorimotor tests (also including forelimb flexion, forelimb placing, accelerated roared and adhesive removal tests) [42, 43]. The test is a measure of symmetry with animals undergoing brain injury, such as ischemia, displaying various asymmetry degrees by consistently turning to the left or right. We found that mice subjected to BCAO and receiving hG-CSF gene for 4- and 7-days, showed significantly less asymmetric turning than BCAO mice with no hG-CSF gene (^#^*p* < 0.05/^##^*p* < 0.01; Fig. 9i, j). This suggests improved neurological deficits by hG-CSF gene therapy. Interestingly, at 7 days, asymmetry was reduced to sham mice’s level by the hG-CSF gene (Fig. 9f) which indicates that the longer exposure to constitutively expressed hG-CSF could result in more considerable improvement in behavior.

Locomotor activity in an open field area such as the force actometer chamber is used as a behavioral paradigm for testing functionality after ischemic brain injury. We examined mice 4- and 7-days post-BCAO and found that mice treated with the AAV-CMV-hG-CSF gene showed relatively high locomotor activity at the first 20–30 min during the 60 min test (Fig. 9k, l). This suggests that the AAV-delivered transgene enhanced neuroprotection, as demonstrated by improved locomotory activity.

## Discussion

Gene therapy is emerging as an exciting and effective therapeutic strategy applied to several neurological disorders and neurodegenerative diseases. The AAV serotype 2 (AAV2) viral vector used in this current study is commonly used for gene delivery in rodent models of stroke and neurodegeneration [44, 45]. In translating gene therapy to human studies/clinical trials, it is imperative to assess any viral vector’s immune response. Several clinical studies with AAV2 indicate that immune responses would impact the efficacy of the gene product rather than the overall safety profile and that in general AAV immunogenicity is affected by the target organ, route of delivery and viral dosage. The central nervous system (CNS), including the eye, have adaptations that limit immune responses compared to peripheral organs such as the liver and heart [46, 47]. The direct delivery in a small dose of AAV2 viral into the brain of Parkinson’s disease patients [48, 49] and the eyes of patients with retinal dystrophy [50, 51] exhibited little or no immune response to the viral vector.

This present study results revealed that the method of introducing a single dose (3 × 10^9^ pfu; 1.5 µl) of the scAAV2 vector carrying the hG-CSF gene into the conjunctival sac of the eye is a reliable approach for delivering sustained hG-CSF into the brain. This result validates our previous study that demonstrated MRI based detection of mRNA for hG-CSF cDNA after eye drop AAV-CMV-hG-CSF administration [26]. In using the BCAO model of global ischemia, we observed in this study the effect of the hG-CSF gene in brain areas supplied by the common carotid artery; striatum and overlying cortex (frontal brain regions) and hippocampus and thalamus (middle brain regions). This study demonstrated that hG-CSF gene therapy delivered by the eye drop method is protective against ER stress-induced apoptosis, mitochondrial stress and autophagy, in the frontal and middle brain regions of the ischemic brain. The protective effect of the single dose of hG-CSF gene therapy was significant at 4 days and 7 days after ischemia, evident from functional improvements determined by corner test and locomotor activity. This evidence is further supported by our previous study on the protective mechanism of the G-CSF protein [52] and provided added credence to the efficacy of hG-CSF gene therapy.

The beneficial effects of G-CSF are mediated by the specific G-CSF receptor. On binding to its receptor, G-CSF activates intracellular pathways, the major pathway being the PI3K/AKT pathway [20]. Activated AKT promotes neuroprotection in the ischemic brain [53, 54] pAkt inhibits several apoptotic substrates, such as caspase 9, and Bad [55], as well as increasing the expression level of Bcl-2, Bcl-xL (anti-apoptotic proteins) and decreases Bax expression level (pro-apoptotic protein) [56]. Here we demonstrated that hG-CSF gene markedly upregulated pAKT 4- and 7-days post-ischemia, indicating that the expressed hG-CSF gene elicits neuroprotection, at least in part, via the PI3K/AKT pathway. Our data also demonstrated that treatment with hG-CSF gene increases the ratio of Bcl2:Bax mRNA and the ratio of Bcl2:Bax protein expression. This observed increase of Bcl2:Bax ratio increases cell survival and could be due to the hG-CSF-induced increase of pAKT.

Perturbation of ER homeostasis by an ischemic insult leads to the accumulation of unfolded proteins (ER stress). ER stress initiates the UPR. The UPR activates the three ER transmembrane sensors; PERK, IRE1 and ATF6, functioning to remove or unfold the defected proteins [57]. In failing to restore ER homeostasis, the UPR activates apoptotic pathways, leading to cell death. All three ER membrane sensors (PERK, IRE1 and ATF6) have downstream players (ATF4, XBP1 and cleaved ATF6, respectively) that induce the pro-apoptotic transcription factor; CHOP. Several lines of study have reported CHOP involvement in the apoptosis of neurons, for example, CHOP^−/−^mice are more resistant to hypoxia reoxygenation-induced neuronal cell death than those from wild-type animals following bilateral common carotid occlusion [58] and ischemic postconditioning protects the brain from ischemia/reperfusion (I/R) injury by decreasing the activation of CHOP at 24 h. of reperfusion [59]. CHOP induces apoptosis via direct inhibition of Bcl-2 transcription and induction of several pro-apoptotic proteins, including Bim and Bax expression [60]. Here we observed that global ischemia increases pIRE1 (the active form of IRE1) and its downstream effector XBP1, as well as ATF4, cleaved ATF6 and CHOP. The upregulation of these ER stress-related proteins verify that global ischemia induces ER stress, making our findings consistent with previous reports [61]. Treatment with the hG-CSF gene drastically reversed the upregulated expression of pIRE1, XBP1, ATF4, cleaved ATF6 and CHOP at 4- and 7-days in the frontal and middle brain sections. As seen in this study, the downregulation of CHOP by hG-CSF gene transfer inevitably results in the upregulation of Bcl2 and downregulation of Bax, due to the functional relation of CHOP transcription of these proteins.

An additional ER stress-induced apoptotic mechanism involves caspase 12. Caspase 12 is activated after permanent and transient middle cerebral artery occlusion and many caspases 12 positive cells exhibit DNA fragmentation [23, 62]. In unstressed cells, tumor necrosis factor receptor-associated factor 2 (TRAF2) formed a stable complex with procaspase12 at the ER membrane. During ER stress, pIRE1 activates TRAF2 downstream and TRAF2 releases procaspase 12, enabling it to oligomerize, which aids in procaspase 12 being activated [63]. Cleaved/activated caspase 12 translocate from the ER to the cytosol where it directly cleaves caspase 9, which in turn activates the effector caspase 3 [11]. Here we observed a reduction of activated caspase 12 (cleaved caspase 12). The observed reduction of pIRE1 by the expressed hG-CSF gene could account for the reduced level of activated caspase 12 since diminished pIRE1 levels would enhance the inhibition of TRAF2 dissociation from procaspase 12.

A typical sensor of the ER intraluminal environment is GRP78. It is a calcium-binding protein chaperone in the ER lumen. During an ischemic injury, the level of GRP78 rises. This rise in GRP78 expression is an attempt by the cell to re-establish proper folding of the accumulated unfolded protein in the ER as well as to sequester ER calcium [Ca^2+^] _ER_ due to the decreasing [Ca^2+^,]_ER_ [64]. We observed that hG-CSF gene treatment markedly reverses the increased expression of GRP78 in global ischemia. Since GRP78 is an ER intraluminal senor, its reduction by the hG-CSF gene indicates that the ER is regaining its normal homeostasis.

Recent studies have revealed that a fraction of cytosolic Bcl2, Bax/Bak is located at the ER membrane and that pro-apoptotic Bax/Bak modulates the UPR by directly interacting with IRE1 [65]. Furthermore, Bax/Bak increases the efflux of (Ca^2+^) ER through the inositol 1,4,5-trisphosphate receptors (IP3 Rs), by direct interference of this receptor channel [66]. These two activities of Bax/Bak at the ER provide a pro-apopotic feedforward loop. Since Bcl2 antagonizes Bax/Bak, our data indicate that hG-CSF gene treatment could counteract the pro- apoptotic feedforward loop due to the upregulation of Bcl2 by the expressed hG-CSF gene. Intriguingly, IP3 Rs are located in mitochondria ER-associated membranes (MAMs). This close association with the mitochondria facilities a rapid transfer of calcium from the ER to the mitochondria [67]. An overload of ER-derived calcium in the mitochondria interferes with the mitochondria’s dynamics and upregulates the mitochondrial fission protein, DRP1 which contributes to excessive fragmentation and subsequent apoptosis [36]. The fusion protein, OPA1, tends to counteract the apoptotic effect of DRP1, thereby preserving the mitochondria. In this study, hG-CSF gene treatment preserved the mitochondria from fragmentation during global ischemia by downregulating DRP1 while upregulating OPA1 expression.

Both ER stress and mitochondrial dysfunction inevitably proteinate autophagy (“self-digesting”) [68]. While autophagy is initially beneficial in digesting damaged cellular components and recycling needed cellular molecules, a surge in autophagy initiation and impeded autophagosome flux/processing during the reperfusion stage of an ischemic injury promotes autophagic cell death [41, 69, 70]. Beclin 1 is an essential autophagic initiator that is upregulated during reperfusion and contributes to autophagic cell death [18, 71]. The expression of Beclin 1 is modulated via the pAKT-Bcl2-Beclin 1 pathway [72]. The detrimental autophagic activation mediated by Beclin 1 is correlated with Bcl2 downregulation since Bcl2 inhibits Beclin 1 by binding to the BH3 only domain of Beclin 1 [73]. We observed the upregulation of Beclin 1 at 4- and 7-days post-global ischemia. However, Beclin 1’s detrimental effect would be counteracted by the hG-CSF gene-mediated upregulation of Bcl2. In addition to Beclin 1 initiating autophagy, the level of LC3-ll (the autophagic vesicle-associated form of LC3) indicates the amount of autophagosomes formed. Meanwhile, 62 links ubiquitinated aggregates for destruction in autophagosomes and is degraded during autophagic flux/autophagosome processing [37, 74]. Therefore, the stimulation of autophagosome processing/flux (which involves the formation of autophagosomes, autophagosome-lysosome fusion and final degradation) would potentiate LC3-ll while depleting p62 and other autophagic substrates. On the other hand, impaired autophagic clearance result in accumulated p62 and cell death [39, 40]. The potentiation of Beclin 1 and LC3-ll in this study indicated an increase in autophagy initiation and autophagosome formation at 4- and 7-days post global ischemia/reperfusion. We also demonstrated that global ischemia/reperfusion impeded the autophagic flux, evident by the accumulation of p62. Our data is supported by several lines of evidence [41, 75]. Cui et al. evaluated autophagic flux in cortical neurons by assessing LC3-ll and p62 in the presence of chloroquine (an inhibitor of autophagosome-lysosome formation) and found increased autophagosome-bound LC3-II and p62; resulting in neuronal death [75]. In this current study, we demonstrated hG-CSF gene transfer not only attenuated excessive autophagy initiation observed by diminished Beclin 1 but also reduced LC3-ll and p62, therefore lowering autophagosome formation as well as simulating autophagic flux, which in total, favors cell survival from autophagic death.

The neuroprotective effect of hG-CSF gene therapy in the frontal and middle brain sections would translate to protecting various populations of neurons in these brain regions. GABA, the CNS’s main inhibitory neurotransmitter is synthesized in GABAergic neurons from glutamate by the two isoforms of GAD; GAD65 and GAD67. GAD67 is present throughout the cytosol, whereas GAD65 preferentially localizes in axonal terminals [76, 77]. GABA neurotransmission activity counteracts hyperexcitation in several neurological disorders including epilepsy, anxiety, Alzheimer’s disease, Parkinson’s disease and Huntington’s disease [78]. Several studies have reported a decrease in GABA concentration in animals and humans suffering an ischemic attack and that there is a loss of GABAergic neurons and GAD protein expression in chronic ischemic rats [79]. Therefore, the GABAergic system could be a potential therapeutic target in cerebral ischemia. Here we observed that hG-CSF gene expression rescued GABAergic neurons in vulnerable brain regions such as the cortex and CA1 and CA3 of the hippocampus. The increased number of GABAergic neurons with hG-CSF gene expression is evident by the increase of both GAD65 mRNA and protein expression. The underlying mechanism of the increase in GABAergic neurons could be due to both neuroprotection and neurogenesis. This aspect of the study is now under investigation.

Previous studies employing rodent models of transient global ischemia and permanent global ischemia have indicated that the number of ChAT positive neurons decreases in the basal forebrain and the hippocampus. Such decreases could be reversed by treatment with neuroprotective agents, concomitant with the reversal of behavioral deficits [80]. Similarly, in the current study, we found decreased numbers of ChAT positive neurons in the basal forebrain at 4 days following transient BCAO with reversal of these decreases resulting from neuroprotective and or neurogenesis effects of hG-CSF gene transfer as discussed above for the GABA system.

The protective effect of hG-CSF gene therapy is reflected in improved functional behavior at 4 days and 7 days post-BCAO. Interestingly, locomotor activity improved for the first 20 min for the hG-CSF gene group at 4 days. Improved locomotion for the first 20 min was enhanced for a longer time period of 30 min at 7 days post BCAO This improved behavior at 7 days over 4 days indicates that the longer constitutive expression of the hG-CSF protein by the hG-CSF gene boosted protection, as reflected in improved behavior. In our previous study [81], we have demonstrated neuronal protection by G-CSF protein in hypoxia–ischemia animal model was in part due to its anti-inflammatory action since the expression levels of pro-inflammatory cytokines, TNF-α and IL-1ß were significantly decreased in the G-CSF protein treated group, while the anti-inflammatory cytokine, IL-10, expression level was increased. Our ongoing study of the mechanistic actions of hG-CSF gene therapy will include anti-inflammatory effect, pro-angiogenesis and pro-neurogenesis mechanisms.

Although the global ischemic (transient BCAO) model used in this study does not provide a complete cessation of blood flow to the entire brain compared to other models, such as ventricular fibrillation/defibrillation, the transient BCAO model has however, been successfully used in several studies to simulate forebrain ischemic pathology of cardiopulmonary arrest/resuscitation (CPR) [82, 83]. The transient BCAO model is very applicable to small animals such as the mice, which makes the model suitable for chronic survival studies. Besides, the use of BCAO application in transgenic mice facilitates the manipulation of gene expression, providing an excellent approach to study mechanisms of forebrain global ischemia–reperfusion injury and neuroprotection.

## Conclusion

Currently, no pharmacological agents have been proven to effectively improve neurological outcomes in hospital-discharged survivors of a cardiac arrest. In this study, we demonstrated the efficacy of human G-CSF gene therapy in re-establishing ER homeostasis, preserving mitochondria dynamics and reducing autophagy’s detrimental action in a mouse model of transient global ischemia. The single administration of the hG-CSF gene provides a sustained expression of the hG-CSF protein, thereby overcoming the 4-h half-life limitation of plasma hG-CSF. Furthermore, our eye drop method of administering the hG-CSF gene is noninvasive. However, translational of AAV2-CMV-hG-CSF gene therapy to stroke trial could be challenging due to limited information available for G-CSF gene therapy using the AAV-viral vector as a delivery vehicle. Recent approval from the FDA for AAV-delivered gene therapy for a rare eye disease namely, Leber congenital amaurosis [84] could serve as a model for using AAV-hG-CSF gene therapy for clinical use in stroke as we report here.

## Supplementary information

**Additional file 1: Fig. S1.** Local cerebral blood flow (LCBF). Laser Doppler Flowmeter (LDF) recording of LCBF: (a) sham-operated mouse showing marginal change in blood flow (b) AAV-CMV-GFP mouse pre- BCAO (mean baseline flow 94.31 PU), during 30mins BCAO (mean 27.43PU,) and reperfusion (mean 154.77 PU). (c) AAV-CMV-hG-CSF mouse pre- BCAO (mean baseline flow 81.99 PU), during 30mins BCAO (mean 10.39 PU) and reperfusion (mean 110.61 PU). (d) Quantitative bar graph of LCBF during 30 mins BCAO. Pre-BCAO, mice were randomly assigned to be treated with either AAV-CMV-hG-CSF (n = 21) or AAV-CMV-GFP (n=27). Sham operated mice (n=16) were not subjected to BCAO. Data were presented as percentage (%) value of baseline LCBF. Values are mean ± SEM. * * * P < 0.001 vs vs sham-operated mice. One-way ANOVA, post hoc Tukey test.

**Additional file 2: Fig. S2.** Representative example of western whole blot. Western blot of GRP78 and loading control at 4-day post-BCAO. First lane shows protein marker. Lanes 1-3 represents the front (striatum and overlying cortex) region of the brain. Lane 1- Sham, lane 2 – AAV-CMV-GFP and lane 3 – AAV-CMV- hG-CSF. Lanes 4 – 6 represents the middle (Hippocampus) region of the brain. Lane 4- Sham, lane 5– AAV-CMV-GFP and lane 6 – AAV-CMV- hG-CSF. Molecular weights of GRP78 and GAPDH 78 kDa and 37 kDa respectively. Densitometry is taken as quantitative intensity of bands and normalized against GAPDH.

**Additional file 3: Table S1.** List of Antibodies used for Western Blotting

## Data Availability

The datasets used and/or analyzed during the current study are available from the corresponding author on reasonable request.

## Abbreviations

AKT/PKB: Protein kinase B
ATF4: Activating transcription factor 4
ATF6: Activating transcription factor 6
AAV: Adeno-associated virus
BAX: Bcl-2 antagonist/killer
BCAO: Bilateral common carotid artery occlusion
BCL2: B cell lymphoma 2
CA1: Cornu Ammonis 1
CA2: Cornu Ammonis 2
CA3: Cornu Ammonis 3
Caspase 12: Cysteine aspartic acid protease 12
CCA: Common carotid artery
CMV: Cytomegalovirus
CHOP: C/EBP homologous protein
CPR: Cardiopulmonary resuscitation
DRP1: Dynamin-related protein 1
ER: Endoplasmic reticulum
ERAD: ER-associated protein degradation
FDA: Food and Drug Administration
FPA: Force-plate actometer
GAD: Glutamic acid decarboxylase
GAD65: Glutamic acid decarboxylase 65
GAD67: Glutamic acid decarboxylase 67
GAPDH: Glyceraldehyde-3-phosphate dehydrogenase
G-CSF: Granulocyte-colony stimulating factor
GFP: Green fluorescent protein
GABA: Gamma-aminobutyric acid
GRP78: Glucose-regulated protein 78
hG-CSF: Human granulocyte-colony stimulating factor
IP3R: Inositol trisphosphate receptor
IRE1α: Inositol-requiring protein-1alpha
LCBF: Local cerebral blood flow
LC3: Microtubule-associated protein 1A/1B-light chain 3
LC3-ll: LC3-phosphatidylethanolamine conjugate
MAM: Mitochondria ER-associated membrane
MCAO: Middle cerebral artery occlusion
OPA1: Optic atrophy protein
P62: Sequestosome-1
P-AKT: Phosphorylated AKT
PFU: Plaque-forming unit
PI3K: Phosphatidylinositol-3-kinase
PIRE1α: Phosphorylated Inositol-requiring protein-1alpha
scAAV2: Self-complementary adeno-associated virus stereotype 2
TTC: Unfold protein response
TRAF2: Tumor necrosis factor receptor-associated factor 2
XBP-1: X-Box-binding protein-1

## References

[1] Berger S (2017). Survival from out-of-hospital cardiac arrest: are we beginning to see progress?. J Am Heart Assoc.

[2] Sano R, Reed JC (2013). ER stress-induced cell death mechanisms. Biochim Biophys Acta - Mol Cell Res.

[3] Zhao CQ, Zhang YH, Jiang SD, Jiang LS, Dai LY (2010). Both endoplasmic reticulum and mitochondria are involved in disc cell apoptosis and intervertebral disc degeneration in rats. Age (Omaha).

[4] Zhang Y, Jin Y, Williams TA, Burtenshaw SM, Martyn AC, Lu R (2010). Amino acid deprivation induces CREBZF/Zhangfei expression via an AARE-like element in the promoter. Biochem Biophys Res Commun.

[5] Oyadomari S, Mori M (2004). Roles of CHOP/GADD153 in endoplasmic reticulum stress. Cell Death Differ.

[6] Ayaub EA, Kolb PS, Mohammed-Ali Z, Tat V, Murphy J, Bellaye P-S (2016). GRP78 and CHOP modulate macrophage apoptosis and the development of bleomycin-induced pulmonary fibrosis. J Pathol.

[7] Kropski JA, Blackwell TS (2018). Endoplasmic reticulum stress in the pathogenesis of fibrotic disease. J Clin Invest.

[8] McCullough KD, Martindale JL, Klotz LO, Aw TY, Holbrook NJ (2001). Gadd153 sensitizes cells to endoplasmic reticulum stress by down-regulating Bcl2 and perturbing the cellular redox state. Mol Cell Biol.

[9] Puthalakath H, O’Reilly LA, Gunn P, Lee L, Kelly PN, Huntington ND (2007). ER stress triggers apoptosis by activating BH3-only protein Bim. Cell.

[10] Zou X-J, Yang L, Yao S-L (2012). Endoplasmic reticulum stress and C/EBP homologous protein-induced Bax translocation are involved in angiotensin II-induced apoptosis in cultured neonatal rat cardiomyocytes. Exp Biol Med.

[11] Morishima N, Nakanishi K, Takenouchi H, Shibata T, Yasuhiko Y (2002). An endoplasmic reticulum stress-specific caspase cascade in apoptosis. Cytochrome c-independent activation of caspase-9 by caspase-12. J Biol Chem.

[12] Li Y, Guo Y, Tang J, Jiang J, Chen Z (2014). New insights into the roles of CHOP-induced apoptosis in ER stress structure and properties of C / EBP homologous protein roles of CHOP in ER stress-mediated apoptosis. Acta Biochim Biophys Sin.

[13] Yamaguchi R, Lartigue L, Perkins G, Scott RT, Dixit A, Kushnareva Y (2008). Opa1-mediated cristae opening is Bax/Bak and BH3 dependent, required for apoptosis, and independent of Bak oligomerization. Mol Cell.

[14] Owens K, Park JH, Gourley S, Jones H, Kristian T (2015). Mitochondrial dynamics: cell-type and hippocampal region specific changes following global cerebral ischemia. J Bioenerg Biomembr.

[15] Hu C, Huang Y, Li L (2017). Drp1-dependent mitochondrial fission plays critical roles in physiological and pathological progresses in mammals. Int J Mol Sci.

[16] Yang Z, Klionsky DJ (2010). Eaten alive: a history of macroautophagy. Nat Cell Biol.

[17] Maiuri MC, Zalckvar E, Kimchi A, Kroemer G (2007). Self-eating and self-killing: crosstalk between autophagy and apoptosis. Nat Rev Mol Cell Biol.

[18] Qian X, Li X, Cai Q, Zhang C, Yu Q, Jiang Y (2017). Phosphoglycerate kinase 1 phosphorylates beclin1 to induce autophagy. Mol Cell.

[19] Sevimli S, Diederich K, Strecker JK, Schilling M, Klocke R, Nikol S (2009). Endogenous brain protection by granulocyte-colony stimulating factor after ischemic stroke. Exp Neurol.

[20] Schabitz WR, Kolimar R, Schwaninger M, Juettler E, Bardutzky J, Scholzke MN (2003). Neuroprotective effect of granulocyte colony-stimulating factor after focal cerebral ischemia. Stroke.

[21] Zhao L-R, Navalitloha Y, Singhal S, Mehta J, Piao C-S, Guo W-P (2007). Hematopoietic growth factors pass through the blood–brain barrier in intact rats. Exp Neurol.

[22] Gibson CL, Bath PM, Murphy SP (2005). G-CSF reduces infarct volume and improves functional outcome after transient focal cerebral ischemia in mice. J Cereb Blood Flow Metab.

[23] Menzie-Suderam JM, Mohammad-Gharibani P, Modi J, Ma Z, Tao R, Prentice H (2018). Granulocyte-colony stimulating factor protects against endoplasmic reticulum stress in an experimental model of stroke. Brain Res.

[24] England TJ, Abaei M, Auer DP, Lowe J, Jones DRE, Sare G (2012). Granulocyte-colony stimulating factor for mobilizing bone marrow stem cells in subacute stroke: the stem cell trial of recovery enhancement after stroke 2 randomized controlled trial. Stroke.

[25] Ren J, Chen YI, Liu CH, Chen P-C, Prentice H, Wu J-Y (2016). Noninvasive tracking of gene transcript and neuroprotection after gene therapy. Gene Ther.

[26] Julius Speetzen L, Endres M, Kunz A (2013). Bilateral common carotid artery occlusion as an adequate preconditioning stimulus to induce early ischemic tolerance to focal cerebral ischemia. J Vis Exp.

[27] Kramer M, Dang J, Baertling F, Denecke B, Clarner T, Kirsch C (2010). TTC staining of damaged brain areas after MCA occlusion in the rat does not constrict quantitative gene and protein analyses. J Neurosci Methods.

[28] Mohammad-Gharibani P, Modi J, Menzie J, Genova R, Ma Z, Tao R (2014). Mode of action of S-methyl-N, N-diethylthiocarbamate sulfoxide (DETC-MeSO) as a novel therapy for stroke in a rat model. Mol Neurobiol.

[29] Fowler SC, Birkestrand BR, Chen R, Moss SJ, Vorontsova E, Wang G (2001). A force-plate actometer for quantitating rodent behaviors: illustrative data on locomotion, rotation, spatial patterning, stereotypies, and tremor. J Neurosci Methods.

[30] Liu CH, Huang S, Kim YR, Rosen BR, Liu PK (2007). Forebrain ischemia-reperfusion simulating cardiac arrest in mice induces edema and DNA fragmentation in the brain. Mol Imaging.

[31] Meuer K, Pitzer C, Teismann P, Kruger C, Goricke B, Laage R (2006). Granulocyte-colony stimulating factor is neuroprotective in a model of Parkinson’s disease. J Neurochem.

[32] Shamas-Din A, Kale J, Leber B, Andrews DW (2013). Mechanisms of action of Bcl-2 family proteins. Cold Spring Harb Perspect Biol.

[33] Nakagawa T, Zhu H, Morishima N, Li E, Xu J, Yankner BA (2000). Caspase-12 mediates endoplasmic-reticulum-specific apoptosis and cytotoxicity by amyloid-β. Nature.

[34] Schneider A, Kruger C, Steigleder T, Weber D, Pitzer C, Laage R (2005). The hematopoietic factor G-CSF is a neuronal ligand that counteracts programmed cell death and drives neurogenesis. J Clin Invest.

[35] Pradeep H, Sharma B, Rajanikant GK (2014). Drp1 in ischemic neuronal death: an unusual suspect. Curr Med Chem.

[36] Flippo KH, Gnanasekaran A, Perkins GA, Ajmal A, Merrill RA, Dickey AS (2018). AKAP1 protects from cerebral ischemic stroke by inhibiting Drp1-dependent mitochondrial fission. J Neurosci.

[37] Kabeya Y, Mizushima N, Ueno T, Yamamoto A, Kirisako T, Noda T (2000). LC3, a mammalian homologue of yeast Apg8p, is localized in autophagosome membranes after processing. EMBO J.

[38] Kabeya Y (2004). LC3, GABARAP and GATE16 localize to autophagosomal membrane depending on form-II formation. J Cell Sci.

[39] Bjørkøy G, Lamark T, Pankiv S, Øvervatn A, Brech A, Johansen T (2009). Chapter 12 monitoring autophagic degradation of p62/SQSTM1. Methods Enzymol.

[40] Pankiv S, Clausen TH, Lamark T, Brech A, Bruun J-A, Outzen H (2007). p62/SQSTM1 binds directly to Atg8/LC3 to facilitate degradation of ubiquitinated protein aggregates by autophagy. J Biol Chem.

[41] Ma X, Liu H, Foyil SR, Godar RJ, Weinheimer CJ, Hill JA (2012). Impaired autophagosome clearance contributes to cardiomyocyte death in ischemia/reperfusion injury. Circulation.

[42] Schaar KL, Brenneman MM, Savitz SI (2010). Functional assessments in the rodent stroke model. Exp Transl Stroke Med.

[43] Zhang L, Schallert T, Zhang ZG, Jiang Q, Arniego P, Li Q, Lu M, Chopp M (2002). A test for detecting long-term sensorimotor dysfunction in the mouse after focal cerebral ischemia. J Neurosci Methods.

[44] Li Z-J, Wang R (2008). rAAV vector-mediated gene therapy for experimental ischemic stroke. Neurol India.

[45] Hocquemiller M, Giersch L, Audrain M, Parker S, Cartier N (2016). Adeno-associated virus-based gene therapy for CNS diseases. Hum Gene Ther.

[46] Enzmann G, Kargaran S, Engelhardt B (2018). Ischemia-reperfusion injury in stroke: impact of the brain barriers and brain immune privilege on neutrophil function. Ther Adv Neurol Disord.

[47] Engelhardt B, Vajkocz P, Weller R (2017). The movers and shapers in immune privilege of the CNS. Nat Immunol.

[48] Kaplitt MG, Feigin A, Tang C, Fitzsimons HL, Mattis P, Lawlor PA (2007). Safety and tolerability of gene therapy with an adeno-associated virus (AAV) borne GAD gene for Parkinson’s disease: an open label, phase I trial. Lancet.

[49] Marks WJ, Bartus RT, Siffert J, Davis CS, Lozano A, Boulis N (2010). Gene delivery of AAV2-neurturin for Parkinson’s disease: a double-blind, randomised, controlled trial. Lancet Neurol.

[50] Simonelli F, Maguire AM, Testa F, Pierce EA, Mingozzi F, Bennicelli JL (2010). Gene therapy for Leber’s congenital amaurosis is safe and effective through 1.5 years after vector administration. Mol Ther.

[51] Bennett J, Wellman J, Marshall KA, McCague S, Ashtari M, DiStefano-Pappas J (2016). Safety and durability of effect of contralateral-eye administration of AAV2 gene therapy in patients with childhood-onset blindness caused by RPE65 mutations: a follow-on phase 1 trial. Lancet.

[52] Modi J, Menzie-Suderam J, Xu H, Trujillo P, Medley K, Marshall ML, Tao R, Prentice H (2020). Mode of action of granulocyte-colony stimulating factor (G-CSF) as a novel therapy for stroke in a mouse model. J Biomed Sci.

[53] Kilic E, Kilic Ü, Soliz J, Bassetti CL, Gassmann M, Hermann DM (2005). Brain-derived erythropoietin protects from focal cerebral ischemia by dual activation of ERK-1/-2 and Akt pathways. FASEB J.

[54] Lan R, Xiang J, Zhang Y, Wang G-H, Bao J, Li W-W (2013). PI3K/Akt pathway contributes to neurovascular unit protection of Xiao-Xu-Ming decoction against focal cerebral ischemia and reperfusion injury in rats. Evid Based Complement Alternat Med.

[55] Manning BD (2004). Balancing Akt with S6K: implications for both metabolic diseases and tumorigenesis. J Cell Biol.

[56] Guo H, Cui H, Peng X, Fang J, Zuo Z, Deng J (2015). Modulation of the PI3K/Akt pathway and Bcl-2 family proteins involved in chicken’s tubular apoptosis induced by nickel chloride (NiCl_2_). Int J Mol Sci.

[57] Hetz C, Papa FR (2018). The unfolded protein response and cell fate control. Mol Cell.

[58] Tajiri S, Oyadomari S, Yano S, Morioka M, Gotoh T, Hamada JI (2004). Ischemia-induced neuronal cell death is mediated by the endoplasmic reticulum stress pathway involving CHOP. Cell Death Differ.

[59] Yuan Y, Guo Q, Ye Z, Pingping X, Wang N, Song Z (2011). Ischemic postconditioning protects brain from ischemia/reperfusion injury by attenuating endoplasmic reticulum stress-induced apoptosis through PI3K-Akt pathway. Brain Res.

[60] Li Y, Guo Y, Tang J, Jiang J, Chen Z (2014). New insights into the roles of CHOP-induced apoptosis in ER stress. Acta Biochim Biophys Sin (Shanghai).

[61] Xin Q, Ji B, Cheng B, Wang C, Liu H, Chen X (2014). Endoplasmic reticulum stress in cerebral ischemia. Neurochem Int.

[62] Shibata M, Hattori H, Sasaki T, Gotoh J, Hamada J, Fukuuchi Y (2003). Activation of caspase-12 by endoplasmic reticulum stress induced by transient middle cerebral artery occlusion in mice. Neuroscience.

[63] Yoneda T, Imaizumi K, Oono K, Yui D, Gomi F, Katayama T (2001). Activation of Caspase-12, an Endoplastic Reticulum (ER) Resident Caspase, through Tumor Necrosis Factor Receptor-associated Factor 2-dependent Mechanism in Response to the ER Stress. J Biol Chem.

[64] Krebs J, Agellon LB, Michalak M (2015). Ca2+ homeostasis and endoplasmic reticulum (ER) stress: An integrated view of calcium signaling. Biochem Biophys Res Commun.

[65] Hetz C, Bernasconi P, Fisher J, Lee A-H, Bassik MC, Antonsson B (2006). Proapoptotic BAX and BAK modulate the unfolded protein response by direct interaction with IRE1alpha. Science..

[66] Pihán P, Carreras-Sureda A, Hetz C (2017). BCL-2 family: integrating stress responses at the ER to control cell demise. Cell Death Differ.

[67] Phillips MJ, Voeltz GK (2016). Structure and function of ER membrane contact sites with other organelles. Nat Rev Mol Cell Biol.

[68] Dong Y, Undyala VV, Gottlieb RA, Mentzer RM, Przyklenk K (2010). Review: autophagy: definition, molecular machinery, and potential role in myocardial ischemia-reperfusion injury. J Cardiovasc Pharmacol Ther.

[69] Duan Q, Yang W, Jiang D, Tao K, Dong A, Cheng H (2016). Spermine ameliorates ischemia/reperfusion injury in cardiomyocytes via regulation of autophagy. Am J Transl Res.

[70] Daniels LJ, Varma U, Annandale M, Chan E, Mellor KM, Delbridge LMD (2019). Myocardial energy stress, autophagy induction, and cardiomyocyte functional responses. Antioxid Redox Signal.

[71] Valentim L, Laurence KM, Townsend PA, Carroll CJ, Soond S, Scarabelli TM (2006). Urocortin inhibits Beclin1-mediated autophagic cell death in cardiac myocytes exposed to ischaemia/reperfusion injury. J Mol Cell Cardiol.

[72] Brady NR, Hamacher-Brady A, Yuan H, Gottlieb RA (2007). The autophagic response to nutrient deprivation in the hl-1 cardiac myocyte is modulated by Bcl-2 and sarco/endoplasmic reticulum calcium stores. FEBS J.

[73] Ciechomska IA, Goemans GC, Skepper JN, Tolkovsky AM (2009). Bcl-2 complexed with Beclin-1 maintains full anti-apoptotic function. Oncogene.

[74] Bjørkøy G, Lamark T, Brech A, Outzen H, Perander M, Øvervatn A (2005). p62/SQSTM1 forms protein aggregates degraded by autophagy and has a protective effect on huntingtin-induced cell death. J Cell Biol.

[75] Cui D, Sun D, Wang X, Yi L, Kulikowicz E, Reyes M (2017). Impaired autophagosome clearance contributes to neuronal death in a piglet model of neonatal hypoxic-ischemic encephalopathy. Cell Death Dis.

[76] Modi J, Prentice H, Wu J-Y (2015). Regulation of GABA neurotransmission by glutamic acid decarboxylase (GAD). Curr Pharm Des.

[77] Wang X, Gao F, Zhu J, Guo E, Song X, Wang S (2014). Immunofluorescently labeling glutamic acid decarboxylase 65 coupled with confocal imaging for identifying GABAergic somata in the rat dentate gyrus-A comparison with labeling glutamic acid decarboxylase. J Chem Neuroanat.

[78] Fontes MAP, Vaz GC, Cardoso TZD, de Oliveira MF, Campagnole-Santos MJ, Dos Santos RAS (2018). GABA-containing liposomes: neuroscience applications and translational perspectives for targeting neurological diseases. Nanomedicine.

[79] Chen C, Zhou X, He J, Xie Z, Xia S, Lu G (2019). The roles of GABA in ischemia-reperfusion injury in the central nervous system and peripheral organs. Oxid Med Cell Longev.

[80] Kim MS, Bang JH, Lee J, Han JS, Baik TG, Jeon WK (2016). Ginkgo biloba L. extract protects against chronic cerebral hypoperfusion by modulating neuroinflammation and the cholinergic system. Phytomedicine.

[81] Dumbuya JS, Chen L, Shu SY, Ma L, Luo W, Li F, Wu JY (2020). G-CSF attenuates neuroinflammation and neuronal poptosis via mTOR/p70S6K signaling pathway in neonatal hypoxia-ischemia rat model. Brain Res..

[82] Murakami K, Kondo T, Kawase M, Chan PH (1998). The development of a new mouse model of global ischemia: focus on the relationships between ischemia duration, anesthesia, cerebral vasculature, and neuronal injury following global ischemia in mice. Brain Res.

[83] Katada R, Akdemir G, Asavapanumas N, Ratelade J, Zhang H, Verkman AS (2014). Greatly improved survival and neuroprotection in aquaporin-4-knockout mice following global. FASEB J.

[84] Smalley E (2017). First AAV gene therapy poised for landmark approval. Nat Biotechnol.

