# Supplementary material for: Granulocyte-colony stimulating factor gene therapy as a novel therapeutics for stroke in a mouse model
Source: J Biomed Sci. 2020 Oct 30;27:99. doi: 10.1186/s12929-020-00692-5 (PMC7596942; doi:10.1186/s12929-020-00692-5)
Supplement: Supplementary file 2 — Additional file 2: Fig. S2. Representative example of western whole blot. Western blot of GRP78 and loading control at 4-day post-BCAO. First lane shows protein marker. Lanes 1-3 represents the front (striatum and overlying cortex) region of the brain. Lane 1- Sham, lane 2 – AAV-CMV-GFP and lane 3 – AAV-CMV- hG-CSF. Lanes 4 – 6 represents the middle (Hippocampus) region of the brain. Lane 4- Sham, lane 5– AAV-CMV-GFP and lane 6 – AAV-CMV- hG-CSF. Molecular weights of GRP78 and GAPDH 78 kDa and 37 kDa respectively. Densitometry is taken as quantitative intensity of bands and normalized against GAPDH. [file 12929_2020_692_MOESM2_ESM.docx]

**TITLE: Granulocyte-colony Stimulating Factor Gene Therapy as a Novel Therapeutics for Stroke in a Mouse Model**

**JOURNAL: Journal of Biomedical Science**

Janet M. Menzie-Suderam^1,2^***** Ph.D; ([jmenzie@fau.edu](mailto:jmenzie@fau.edu)), Jigar Modi^1,3^***** MD, Ph.D; ([jmodi@health.fau.edu](mailto:jmodi@health.fau.edu)), Hongyaun Xu^1^ ; ([hongchou@fau.edu](mailto:hongchou@fau.edu)), Andrew Bent^1^ MS ([abent@fau.edu](mailto:abent@fau.edu)), Paula Trujillo^2^ BS ([ptrujillo2013@fau.edu](mailto:ptrujillo2013@fau.edu)), Kristen Medley^4^ BS ([Kristen.medley@nyumc.org](mailto:Kristen.medley@nyumc.org)) Eugenia Jimenez^1^ MS ([ejimeneza@fau.edu](mailto:ejimeneza@fau.edu)), Jessica Shen^1^ BS ([jshen2013@fau.edu](mailto:jshen2013@fau.edu)), Michael Marshall^5^ DC, AP, Ph.D. ([mlmmlmmlm@aol.com](mailto:mlmmlmmlm@aol.com)); Rui Tao^1^ Ph.D; ([rtao@health.fau.edu](mailto:rtao@health.fau.edu)), Howard Prentice^1,2,3^ Ph.D; ([hprentic@health.fau.edu](mailto:hprentic@health.fau.edu)), Jang-Yen Wu ^1,2,3,^  Ph.D; [jwu@health.fau.edu](mailto:jwu@health.fau.edu)

**Addresses:**

^1^ Department of Biomedical Sciences, Charles E. Schmidt College of Medicine, Florida Atlantic University, Boca Raton, FL 33431, USA.

² Program in Integrative Biology, Florida Atlantic University, Boca Raton, FL 33431

^3^ Complex Systems and Brain Sciences, Florida Atlantic University, Boca Raton, FL

^4^ College of Medicine, New York University, New York, NY 10003

^5^AEURA Trust, 2525 Arapahoe Ave E4-138, Boulder, Colorado 80302, USA

**Co-corresponding Authors:**

Rui Tao: [rtao@health.fau.edu](mailto:rtao@health.fau.edu), Tel: 561-297, Fax: 561-297-2221

Howard Prentice: [hprentic@health.fau.edu](mailto:hprentic@health.fau.edu), Tel: 561-297-0362, Fax: 561-297-2221

Jang-Yen Wu: [jwu@health.fau.edu](mailto:jwu@health.fau.edu), Tel: 561-297-0167, Fax: 561-297-2221

*****Indicates equivalent authorship


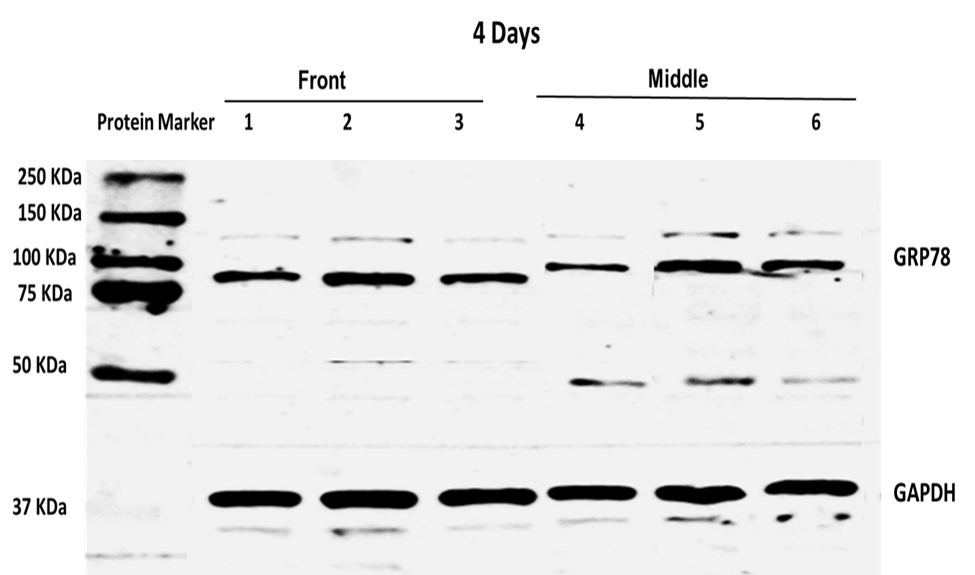
 **Fig. S2 Representative example of western whole blot**

Western blot of GRP78 and loading control at 4-day post-BCAO. First lane shows protein marker. Lanes 1-3 represents the front (striatum and overlying cortex) region of the brain. Lane 1- Sham, lane 2 – AAV-CMV-GFP and lane 3 – AAV-CMV- hG-CSF. Lanes 4 – 6 represents the middle (Hippocampus) region of the brain. . Lane 4- Sham, lane 5– AAV-CMV-GFP and lane 6 – AAV-CMV- hG-CSF. Molecular weights of GRP78 and GAPDH 78 kDa and 37 kDa respectively. Densitometry is taken as quantitative intensity of bands and normalized against GAPDH.
