# Supplementary material for: Granulocyte-colony stimulating factor gene therapy as a novel therapeutics for stroke in a mouse model
Source: J Biomed Sci. 2020 Oct 30;27:99. doi: 10.1186/s12929-020-00692-5 (PMC7596942; doi:10.1186/s12929-020-00692-5)
Supplement: Supplementary file 3 — Additional file 3: Table S1. List of Antibodies used for Western Blotting [file 12929_2020_692_MOESM3_ESM.docx]

**TITLE: Granulocyte-colony Stimulating Factor Gene Therapy as a Novel Therapeutics for Stroke in a Mouse Model**

**JOURNAL: Journal of Biomedical Science**

Janet M. Menzie-Suderam^1,2^***** Ph.D; ([jmenzie@fau.edu](mailto:jmenzie@fau.edu)), Jigar Modi^1,3^***** MD, Ph.D; ([jmodi@health.fau.edu](mailto:jmodi@health.fau.edu)), Hongyaun Xu^1^ ; ([hongchou@fau.edu](mailto:hongchou@fau.edu)), Andrew Bent^1^ MS ([abent@fau.edu](mailto:abent@fau.edu)), Paula Trujillo^2^ BS ([ptrujillo2013@fau.edu](mailto:ptrujillo2013@fau.edu)), Kristen Medley^4^ BS ([Kristen.medley@nyumc.org](mailto:Kristen.medley@nyumc.org)) Eugenia Jimenez^1^ MS ([ejimeneza@fau.edu](mailto:ejimeneza@fau.edu)), Jessica Shen^1^ BS ([jshen2013@fau.edu](mailto:jshen2013@fau.edu)), Michael Marshall^5^ DC, AP, Ph.D. ([mlmmlmmlm@aol.com](mailto:mlmmlmmlm@aol.com)); Rui Tao^1^ Ph.D; ([rtao@health.fau.edu](mailto:rtao@health.fau.edu)), Howard Prentice^1,2,3^ Ph.D; ([hprentic@health.fau.edu](mailto:hprentic@health.fau.edu)), Jang-Yen Wu ^1,2,3,^  Ph.D; [jwu@health.fau.edu](mailto:jwu@health.fau.edu)

**Addresses:**

^1^ Department of Biomedical Sciences, Charles E. Schmidt College of Medicine, Florida Atlantic University, Boca Raton, FL 33431, USA.

² Program in Integrative Biology, Florida Atlantic University, Boca Raton, FL 33431

^3^ Complex Systems and Brain Sciences, Florida Atlantic University, Boca Raton, FL

^4^ College of Medicine, New York University, New York, NY 10003

^5^AEURA Trust, 2525 Arapahoe Ave E4-138, Boulder, Colorado 80302, USA

**Co-corresponding Authors:**

Rui Tao: [rtao@health.fau.edu](mailto:rtao@health.fau.edu), Tel: 561-297, Fax: 561-297-2221

Howard Prentice: [hprentic@health.fau.edu](mailto:hprentic@health.fau.edu), Tel: 561-297-0362, Fax: 561-297-2221

Jang-Yen Wu: [jwu@health.fau.edu](mailto:jwu@health.fau.edu), Tel: 561-297-0167, Fax: 561-297-2221

*****Indicates equivalent authorship

**Table S1 List of Antibodies used for Western Blotting**

| **Antibody** | **Company and Catalogue Number** |
| --- | --- |
| Anti-p62 | Abcam, Cat# ab56416 |
| Anti-GRP78 antibody | Abcam, Cat# ab21685 |
| Anti-ATF4 antibody | Abcam, Cat# ab85049 |
| Anti-Caspase-12 antibody | Abcam, Cat# ab62484 |
| Anti-OPA1 antibody | Abcam, Cat# ab157457 |
| Anti-DRP1 antibody | Abcam, Cat# ab184247 |
| Anti-XBP1 antibody  Anti- IRE1 antibody  Anti- Phospho IRE1 (p-IRE1) | Abcam, Cat# ab37152  Abcam, Cat# ab37073  Abcam, Cat# ab48187 |
| Anti-GAPDH antibody | Cell Signaling Technologies, Cat# 5174S |
| Anti-Akt antibody | Cell Signaling Technologies, Cat# 4691S |
| Anti-Phospho Akt (P-Akt) antibody | Cell Signaling Technologies, Cat# 4060S |
| Anti-Bax antibody | Cell Signaling Technologies, Cat# 2772S |
| Anti-Beclin-1 antibody | Cell Signaling Technologies, Cat# 3738S |
| Anti-G-CSF antibody | Santa Cruz Cat# sc-53292 |
| Anti CHOP/GADD153 antibody | SantaCruz Biotechnology, Cat# sc-793 |
| Anti-Bcl-2 (N-19) antibody | SantaCruz Biotechnology, Cat# sc-492 |
| Anti-ATF6 antibody  Anti-LC3-II antibody | Imgenex, Cat#IMG-273  Santa Cruz Biotechnolgy, Cat# sc-398822 |
| Secondary goat anti-mouse antibody | LI-COR Bioscience, cat#925-32210 |
| Secondary goat anti-rabbit antibody | LI-COR Bioscience, cat#925-32211 |
